# Atrial Fibrillation and Reperfusion Therapy in Acute Ischaemic Stroke Patients: Prevalence and Outcomes—A Comprehensive Systematic Review and Meta-Analysis

**DOI:** 10.3390/neurolint15030065

**Published:** 2023-08-25

**Authors:** Jay Patel, Sonu M. M. Bhaskar

**Affiliations:** 1 Global Health Neurology Lab., Sydney, NSW 2150, Australia; 2 South Western Sydney Clinical Campuses, University of New South Wales (UNSW) Medicine and Health, UNSW Sydney, Sydney, NSW 2170, Australia; 3 Neurovascular Imaging Laboratory, Clinical Sciences Stream, Ingham Institute for Applied Medical Research, Sydney, NSW 2170, Australia; 4 NSW Brain Clot Bank, NSW Health Pathology, Sydney, NSW 2170, Australia; 5 Department of Neurology & Neurophysiology, Liverpool Hospital & South Western Sydney Local Health District (SWSLHD), Sydney, NSW 2170, Australia

**Keywords:** atrial fibrillation, stroke, prevalence, outcomes, reperfusion therapy, thrombolysis, thrombectomy

## Abstract

Atrial fibrillation (AF) significantly contributes to acute ischaemic stroke (AIS), yet its precise influence on clinical outcomes post-intravenous thrombolysis (IVT) and post-endovascular thrombectomy (EVT) has remained elusive. Furthermore, the overall prevalence of AF in AIS patients undergoing reperfusion therapy has not been clearly determined. Employing random-effects meta-analyses, this research aimed to estimate the pooled prevalence of AF among AIS patients undergoing reperfusion therapy, while also examining the association between AF and clinical outcomes such as functional outcomes, symptomatic intracerebral haemorrhage (sICH) and mortality. Studies comparing AF and non-AF patient groups undergoing reperfusion therapy were identified and included following an extensive database search. Forty-nine studies (n = 66,887) were included. Among IVT patients, the prevalence of AF was 31% (Effect Size [ES] 0.31 [95%CI 0.28–0.35], *p* < 0.01), while in EVT patients, it reached 42% (ES 0.42 [95%CI 0.38–0.46], *p* < 0.01), and in bridging therapy (BT) patients, it stood at 36% (ES 0.36 [95%CI 0.28–0.43], *p* < 0.01). AF was associated with significantly lower odds of favourable 90-day functional outcomes post IVT (Odds Ratio [OR] 0.512 [95%CI 0.376–0.696], *p* < 0.001), but not post EVT (OR 0.826 [95%CI 0.651–1.049], *p* = 0.117). Our comprehensive meta-analysis highlights the varying prevalence of AF among different reperfusion therapies and its differential impact on patient outcomes. The highest pooled prevalence of AF was observed in EVT patients, followed by BT and IVT patients. Interestingly, our analysis revealed that AF was significantly associated with poorer clinical outcomes following IVT. Such an association was not observed following EVT.

## 1. Introduction

Atrial fibrillation (AF) is a cardiac arrythmia that holds significant global importance due to its high prevalence, clinical implications, and potentially serious complications [1]. It stands as the most common arrythmia [2], with a prevalence estimated at 0.51%, escalating to 10–17% in individuals aged 80 and above [3]. The burden on public health systems and resources is substantial [1,4,5], primarily due to complications such as acute ischaemic stroke (AIS) [6], a consequence of the stasis-induced thrombus formation within fibrillating atria [7]. These thrombi may embolise to cerebral circulation, leading to AIS [8]. Although approximately 23.7% of AIS or transient ischaemic attack (TIA) patients have underlying AF, AF frequently remains undiagnosed due to limitations of current cardiac monitoring methods [9]. Diagnosis of AF can enable interventions such as prophylactic anticoagulant therapy, often utilizing agents like Vitamin K Antagonists (VKAs) or Non-Vitamin K Oral Anticoagulants (NOACs), potentially preventing a considerable proportion of strokes [10,11]. Notably, the chronic administration of oral anticoagulants has been shown to significantly reduce the risk of ischaemic stroke by up to 64% [12]. Despite the consistent use of these agents or the maintenance of an appropriate International Normalised Ratio (INR), individuals with AF remain susceptible to the potential severity of ischaemic stroke due to the presence of concurrent factors that frequently accompany AF [13,14,15,16]. It is important to acknowledge that the efficacy of these medications lies primarily in preventing embolic events, and they may not comprehensively address a broader spectrum of associated risk factors [16]. Following AIS, patients might undergo reperfusion therapy through intravenous thrombolysis (IVT) or endovascular thrombectomy (EVT) [17]. EVT aims to mechanically remove thrombi [18], whilst IVT strives to dissolve thrombi by cleaving their fibrin network [19,20]. Eligible patients could receive a combination of IVT and EVT, termed bridging therapy (BT) [21].

Reperfusion therapy has become standard in the acute management of ischaemic stroke patients [17]. While various studies have reported on AF prevalence among AIS patients undergoing IVT [22,23,24], EVT [25,26,27] and BT [28,29,30], a comprehensive estimation of the pooled AF prevalence via meta-analysis is still lacking. Comparative analysis of AF prevalence between IVT, EVT and BT will additionally reveal which form of reperfusion therapy is most likely to contain AF patients. Examining regional variations in AF prevalence may further suggest areas where underdiagnosis is more likely [31]. This data is critical to develop evidence-based policy to guide resource allocation for AF screening, stroke prevention and lifestyle-related interventions [5,32]. Despite the use of reperfusion therapy, it is also not clear how AF impacts the effectiveness of these treatments [33,34]. Resolving this uncertainty holds paramount importance in devising optimal treatment strategies, as well as for risk stratification and prognosis communication with patients [35].

This meta-analysis seeks to assess the pooled prevalence of AF in AIS patients receiving reperfusion therapy and the impact of AF on clinical outcomes subsequent to reperfusion therapy. The clinical outcomes encompass 90-day functional status, symptomatic intracerebral haemorrhage (sICH), and 90-day mortality. To address these aspects, our study aims to answer the underlying key questions:

1. What is the prevalence of AF among AIS patients treated with each type of reperfusion therapy?
2. Is there an association between AF and a favourable 90-day functional outcome in AIS patients treated with each type of reperfusion therapy?
3. Does AF correlate with the occurrence of sICH in AIS patients treated with each type of reperfusion therapy?
4. Is AF associated with 90-day mortality in AIS patients treated with each type of reperfusion therapy?

## 2. Methods

### 2.1. Literature Search: Study Identification and Selection

**Figure 1:**
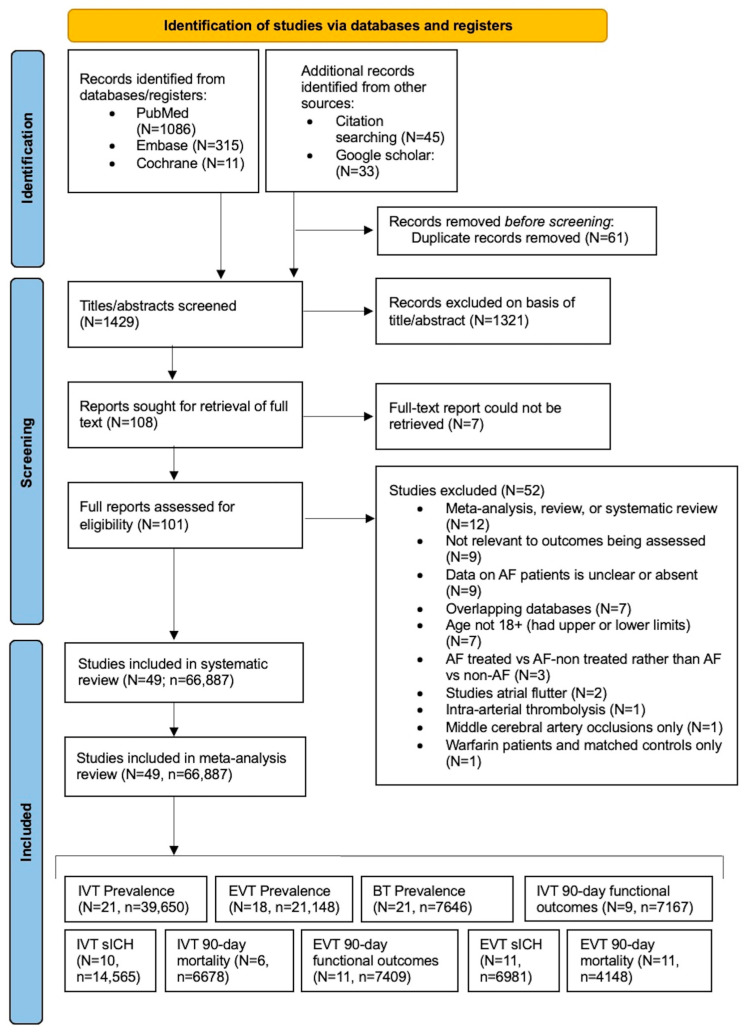
**PRISMA flowchart illustrating the process of study selection.** Abbreviations: N = number of studies, n = total number of patients, AF = atrial fibrillation, IVT = intravenous thrombolysis, EVT = endovascular thrombectomy, BT = bridging therapy, sICH = symptomatic intracerebral haemorrhage.

Studies were retrieved from the databases of PubMed, Embase and Cochrane. This study was conducted in accordance with the Preferred Reporting Items for Systematic Reviews and Meta-Analyses (PRISMA) guidelines and the Meta-analysis of Observational Studies in Epidemiology (MOOSE) checklist (Supplemental Tables S1 and S2). The PRISMA flowchart depicts the number of studies identified, screened, and included in the study (Figure 1). The search was executed on 17 April 2023, with no restrictions on the date of publication. However, our final analysis encompassed IVT studies published from 2005 onwards, and EVT and BT studies published from 2015 onwards. Keywords in the search included “atrial fibrillation”, “ischaemic stroke”, “reperfusion therapy”, “thrombolysis”, “thrombectomy” and “bridging thrombolysis”. Medical Subject Headings (MeSH) terms were used in PubMed to refine the search [36]. Filters were applied to exclude non-English language studies and animal studies. Further records were sourced from citations within retrieved articles and via Google Scholar. For an in-depth understanding of our search strategy, including the keywords and filters, refer to the Supplemental Information (“Search Strategy”).

### 2.2. Inclusion and Exclusion Criteria

Studies were eligible if they met the following criteria: (1) patients diagnosed with AIS; (2) patients aged 18 years and above; (3) consecutive patients who underwent IVT and/or EVT; (4) availability of data on the prevalence of AF; and (5) studies demonstrating robust methodological design (with a minimum sample size of 20 patients). Exclusion criteria comprised (1) animal studies; (2) studies with inaccessible full-text reports; (3) studies published in languages other than English; (4) subsequent studies from the same database; and (5) studies involving intra-arterial thrombolysis.

### 2.3. Data Extraction

The titles and abstracts of all studies from the search were initially screened using EndNote 20.5 (Clarivate, Philadelphia, PA, USA). Studies that clearly did not meet the specified eligibility criteria were excluded at this preliminary stage. Subsequently, full-text reports were obtained for the remaining studies whenever possible. These reports were thoroughly assessed to determine their eligibility for inclusion in our analysis. Meta-analyses, reviews and systematic reviews were excluded at this stage. However, reading the full texts of these studies yielded additional studies for potential inclusion in our analyses and provided valuable insights for the subsequent discussion. The screening process was conducted independently by two researchers, and any discrepancies were resolved through consensus discussions.

Data extraction sheets were prepared to capture the following details: (1) study characteristics (author, year, country, number of centres, study type); (2) reperfusion therapy type (IVT, EVT, or BT); (3) number of patients receiving reperfusion therapy with and without AF; (4) patient demographics (age, male sex, baseline National Institutes of Health Stroke Scale (NIHSS) score); (5) comorbidities (diabetes, lipid disorders, hypertension, coronary artery disease, heart failure, previous stroke/TIA, smoking); and (6) clinical outcomes (90-day functional status, sICH, 90-day mortality). Functional status was assessed using the modified Rankin Scale (mRS) [37], with mRS scores of zero-to-two indicating favourable functional outcomes and scores of three-to-six indicating poor outcomes. The 90-day timepoint was selected for functional outcomes and mortality data, as it serves as a predictive measure of long-term prognosis and is commonly available [38]. The Wan et al. [39] method was employed to approximate the mean and standard deviation (SD) from the median and interquartile range (IQR) values for age and baseline NIHSS where appropriate.

### 2.4. Assessment of Methodological Quality in Included Studies

To ensure methodological rigor, we employed the modified Jadad scale to evaluate the quality of the studies included in the meta-analysis [40]. This assessment focused on elements such as randomisation, blinding, patient withdrawals, inclusion/exclusion criteria clarity, reporting of adverse events, and statistical analysis methods [41]. The detailed evaluation of each study can be found in Supplemental Table S3. Additionally, we considered potential funding bias by examining funding source(s) and author conflicts of interest for any authors. Studies received a bias score: zero indicated low potential for bias, one or two indicated moderate potential, and three suggested high potential.

### 2.5. Statistical Analysis

Statistical analyses were conducted using STATA (Version 13.0, StataCorp, College Station, TX, USA). Meta-analyses were performed if at least four studies were available. For pooled prevalence of AF, we employed the “metaprop” package, utilising a random-effects model with refined 95% confidence intervals (95% CI) using the “cimethod (exact)” and “ftt” commands [42]. Subgroup analyses were conducted based on study type and region.

For the association between AF and clinical outcomes following reperfusion therapy, we used the STATA’s “metan” package to perform meta-analyses and generate forest plots using odds ratios (ORs) as the measure of association. A random effects model was implemented with the DerSimonian–Laird (DL) method [43]. Subgroup analyses compared the prospective and retrospective studies. The “metaninf” package was utilised in STATA to examine changes in the pooled odds ratios if a single study was to be omitted. To assess for publication bias, Egger’s test and funnel plots were used from the “metabias” and “metafunnel” packages. Asymmetry in the funnel plot could indicate publication bias, further verified by the *p*-value from Egger’s test.

Heterogeneity was assessed primarily using the I^2^ statistic, with values categorised as 0–40%, 30–60%, 50–90%, and 75–100% representing low, moderate, substantial, and considerable heterogeneity, respectively [44]. Cochran’s Q test *p*-values were also considered, and between-study variances were estimated using Tau-squared. A significance level of *p* < 0.05 was applied to all analyses in this study [45].

## 3. Results

The initial search across PubMed, Embase and Cochrane databases yielded a total of 1412 records (as outlined in detail in the PRISMA flowchart, Figure 1). Additionally, 78 sources were identified through Google Scholar and by handsearching the references of previous reports. After removing duplicates, the titles and abstract of 1429 studies were screened. Subsequently, 108 full-text reports were assessed, of which 101 could be successfully retrieved. Among these, 52 of these studies were excluded due to non-compliance with inclusion criteria. Finally, a rigorous selection process led to the inclusion of 49 studies for the systematic review and subsequent meta-analysis.

### 3.1. Description of Included Studies

This meta-analysis included 49 studies, comprising a total of 66,887 patients. Twenty-one studies reported on the prevalence of AF in patients receiving IVT [22,23,24,46,47,48,49,50,51,52,53,54,55,56,57,58,59,60,61,62,63], of which thirteen additionally reported on clinical outcomes in patients with AF compared to those without AF [22,23,24,46,47,49,50,53,55,58,59,61,62]. Eighteen studies [25,26,27,33,64,65,66,67,68,69,70,71,72,73,74,75,76,77] reported on the prevalence of AF in patients receiving EVT, of which eleven additionally reported on clinical outcomes in patients with AF compared to those without AF [25,26,27,64,68,69,71,72,73,77,78]. Nine of the EVT studies [25,26,27,33,68,71,72,73,77] also reported on the prevalence of AF in patients receiving BT. An additional nine studies reported on the prevalence of AF in patients receiving BT [28,29,30,79,80,81,82,83,84]. Three studies further reported on clinical outcomes in AF patients compared to non-AF patients receiving BT [28,73,81].

Table 1 presents the clinical characteristics of the 49 studies, encompassing both the total number of patients with AF and the overall total number of patients. This table provides a comprehensive overview of the diversity observed across the studies included in our meta-analysis. Notably, it highlights variations in patient demographics, study design, and geographical distribution. Further description of comorbidities such as hypertension, diabetes, and dyslipidaemia can be found in Table 2. Moreover, Table 3 presents tabulated outcomes of the clinical outcomes of patients, while Table 4 offers a concise summary of the outputs of the meta-analysis.

### 3.2. Prevalence of AF in Patients Treated with IVT

**Figure 2:**
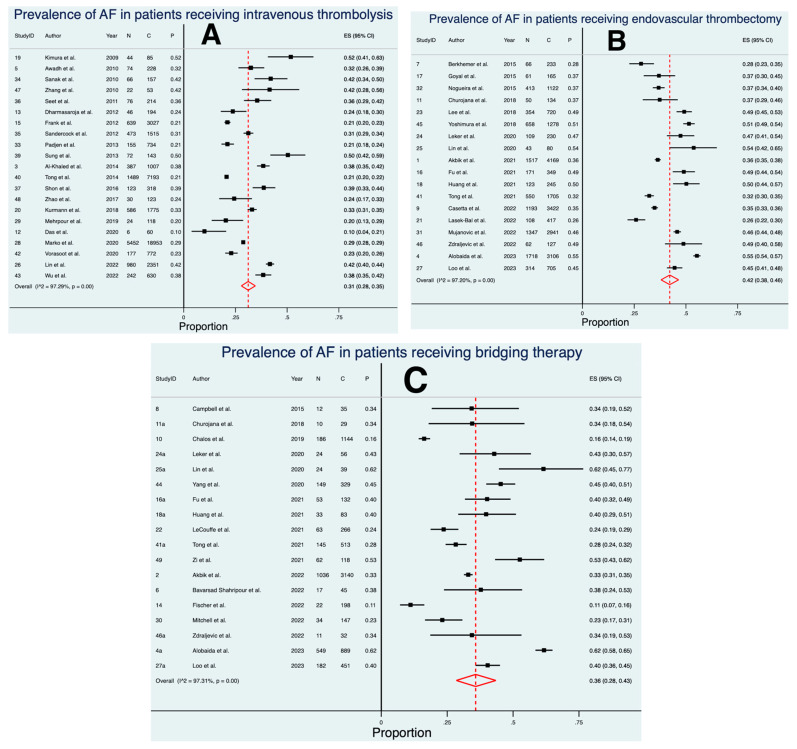
Forest plots of the pooled prevalence of atrial fibrillation in acute ischaemic stroke patients receiving reperfusion therapy. (**A**) intravenous thrombolysis, (**B**) endovascular thrombectomy, and (**C**) bridging therapy. Abbreviations: AF = atrial fibrillation, ES = effect size, CI = confidence interval, N = number of patients with AF, C = total number of patients, P = prevalence.

**Figure 3:**
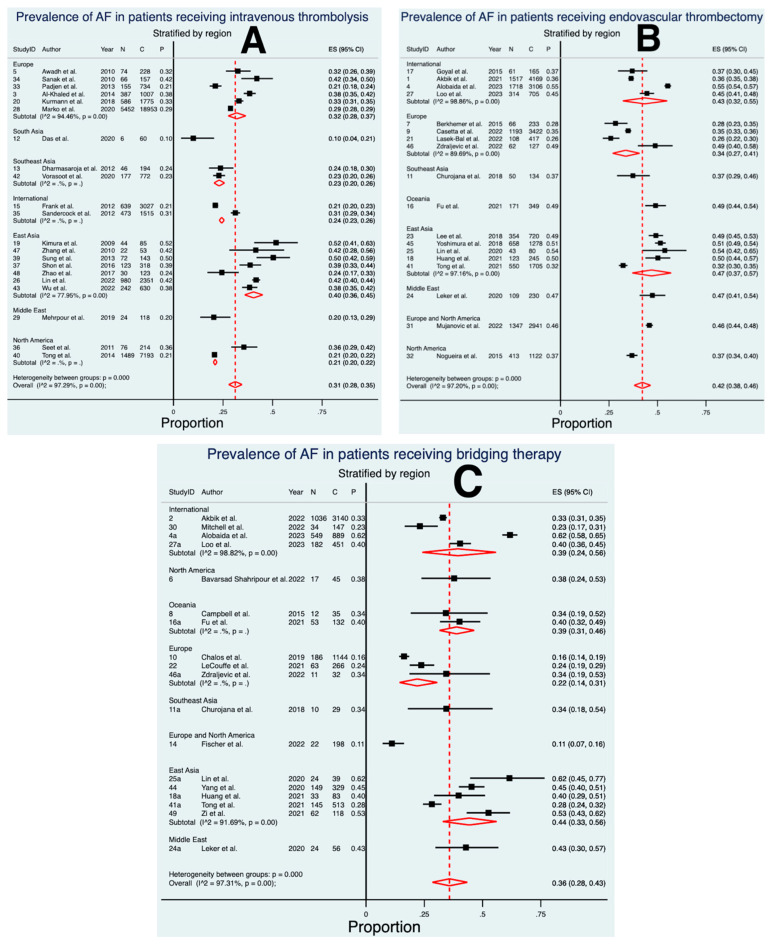
Forest plots of the estimated pooled prevalence of atrial fibrillation in acute ischaemic stroke patients receiving each type of reperfusion therapy, stratified by region. (**A**) intravenous thrombolysis, (**B**) endovascular thrombectomy, and (**C**) bridging therapy. Abbreviations: AF = atrial fibrillation, ES = effect size, CI = confidence interval, N = number of patients with AF, C = total number of patients, P = prevalence.

Twenty-one studies, comprising a total of 39,650 patients, were included in the final meta-analysis for the prevalence of AF in patients receiving IVT (Figure 2 and Figure 3) [22,23,24,46,47,48,49,50,51,52,53,54,55,56,57,58,59,60,61,62,63]. The meta-analysis revealed an estimated prevalence of 31% (ES 0.31 [95% CI 0.28 to 0.35], *p* < 0.01). Considerable heterogeneity was found between the studies (I^2^ = 97.3%, *p* < 0.01). The crude prevalence of AF was 28.2%, which was obtained by dividing the total number of AF patients receiving IVT by the overall cohort of patients receiving IVT in this analysis.

### 3.3. Prevalence of AF in Patients Treated with EVT

Eighteen studies, comprising a total of 21,148 patients, were included in the final meta-analysis for the prevalence of AF in patients receiving EVT (Figure 2 and Figure 3) [25,26,27,33,64,65,66,67,68,69,70,71,72,73,74,75,76,77]. The meta-analysis revealed an estimated pooled prevalence of 42% (ES 0.42 [95% CI 0.38 to 0.46], *p* < 0.01). Considerable heterogeneity was found between the studies (I^2^ = 97.2%, *p* < 0.01). The crude prevalence of AF was 41.9%, which was obtained by dividing the total number of AF patients receiving EVT by the overall cohort of patients receiving EVT in this analysis.

### 3.4. Prevalence of AF in Patients Treated with BT

Eighteen studies, comprising a total of 7646 patients, were included in the final meta-analysis for the prevalence of AF in patients receiving BT (Figure 2 and Figure 3) [25,26,27,28,29,30,33,68,71,72,73,77,79,80,81,82,83,84]. The meta-analysis revealed an estimated pooled prevalence of 36% (ES 0.36 [95% CI 0.28 to 0.43], *p* < 0.01). Considerable heterogeneity was found between the studies (I^2^ = 97.3%, *p* < 0.01). The crude prevalence of AF was 34.2%, which was obtained by dividing the total number of AF patients receiving BT by the overall cohort of patients receiving BT in this analysis.

### 3.5. Association between AF and Favourable 90-Day Functional Outcomes Following IVT

**Figure 4:**
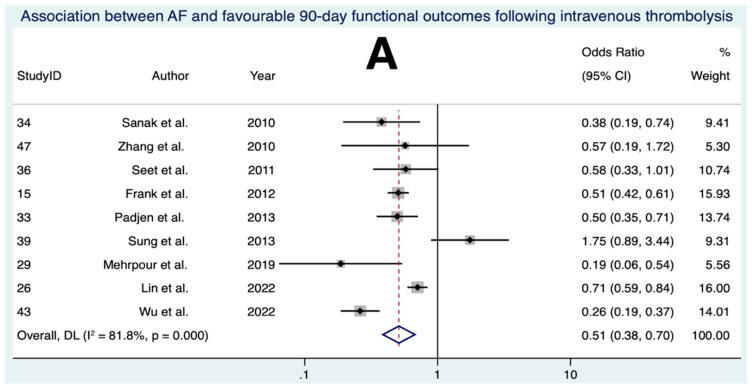

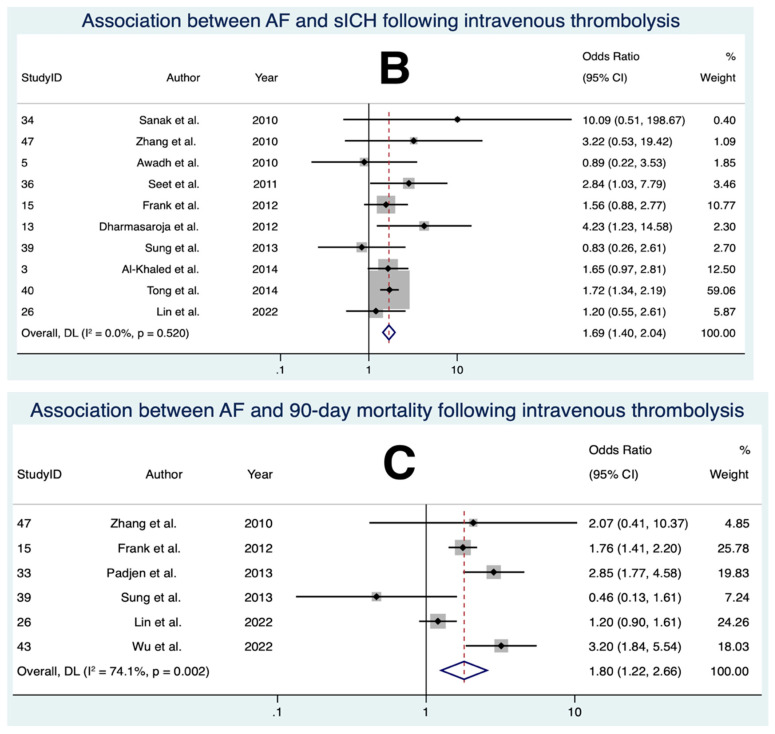
**Forest plots of the association between atrial fibrillation and outcomes following intravenous thrombolysis.** (**A**) association between atrial fibrillation and favourable 90-day functional outcomes. (**B**) association between atrial fibrillation and symptomatic intracerebral haemorrhage. (**C**) association between atrial fibrillation and 90-day mortality. Abbreviations: AF = atrial fibrillation, CI = confidence interval, sICH = symptomatic intracerebral haemorrhage, DL = DerSimonian–Laird.

Nine studies, comprising a total of 7167 patients, were included in the final meta-analysis for the association between AF and favourable 90-day functional outcomes following IVT (Figure 4) [22,23,24,50,53,55,58,61,62]. Overall, the meta-analysis revealed that AF was associated with significantly lower odds of favourable functional outcomes at 90 days following IVT (OR 0.512 [95% CI 0.376 to 0.696], *p* < 0.001). Substantial to considerable heterogeneity was found between the studies (I^2^ = 81.8%, [95% CI < 0.1% to 93.4%], *p* < 0.001). Visual inspection of the funnel plot (Supplemental Figure S1) and the output from Egger’s test (Supplemental Table S4) did not provide evidence of significant publication bias.

### 3.6. Association between AF and sICH Following IVT

Ten studies, comprising a total of 14,565 patients, were included in the final meta-analysis for the association between AF and sICH following IVT (Figure 4) [22,23,46,47,49,50,53,58,59,62]. The definition of sICH used in each study is outlined in Table 3. Overall, the meta-analysis revealed that AF was associated with significantly higher odds of sICH following IVT (OR 1.690 [95% CI 1.400 to 2.039], *p* = 0.851). Low heterogeneity was found between the studies (I^2^ = 0.0% [95% CI < 0.1% to 48.0%], *p* = 0.520). Visual inspection of the funnel plot (Supplemental Figure S1) and the output from Egger’s test (Supplemental Table S4) did not provide evidence of significant publication bias.

### 3.7. Association between AF and 90-Day Mortality Following IVT

Six studies, comprising a total of 6678 patients, were included in the final meta-analysis for the association between AF and 90-day mortality following IVT (Figure 4) [50,53,55,58,61,62]. Overall, the meta-analysis revealed that AF was associated with significantly higher odds of mortality at 90 days following IVT (OR 1.799 [95% CI 1.218 to 2.657], *p* = 0.003). Substantial heterogeneity was found between the studies (I^2^ = 74.1% [95% CI < 0.1% to 91.5%], *p* = 0.002). Visual inspection of the funnel plot (Supplemental Figure S1) and the output from Egger’s test (Supplemental Table S4) did not provide evidence of significant publication bias.

### 3.8. Association between AF and Favourable 90-Day Functional Outcomes Following EVT

**Figure 5:**
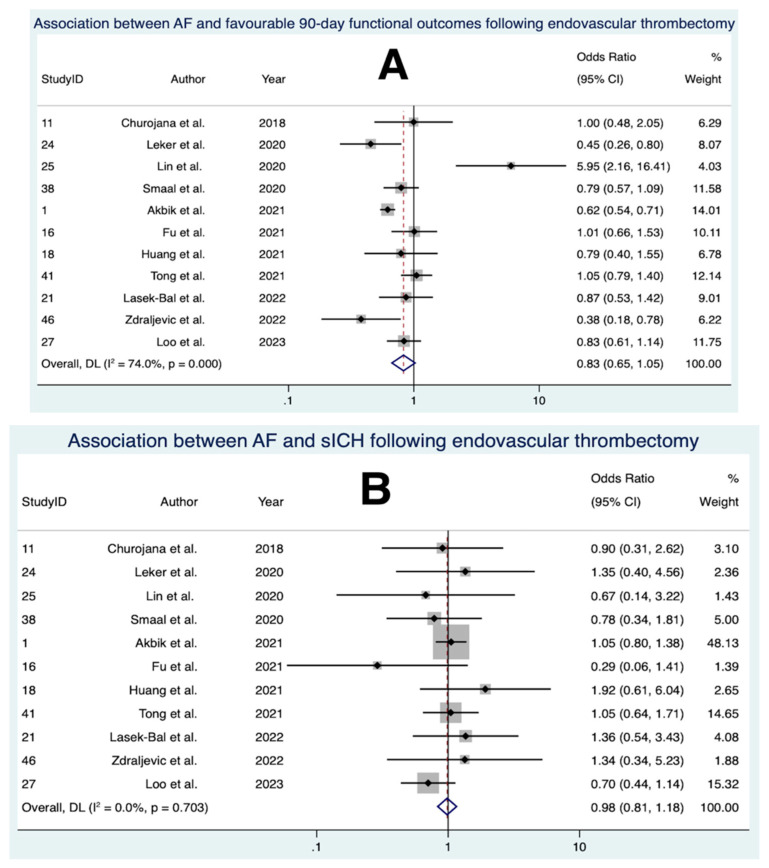

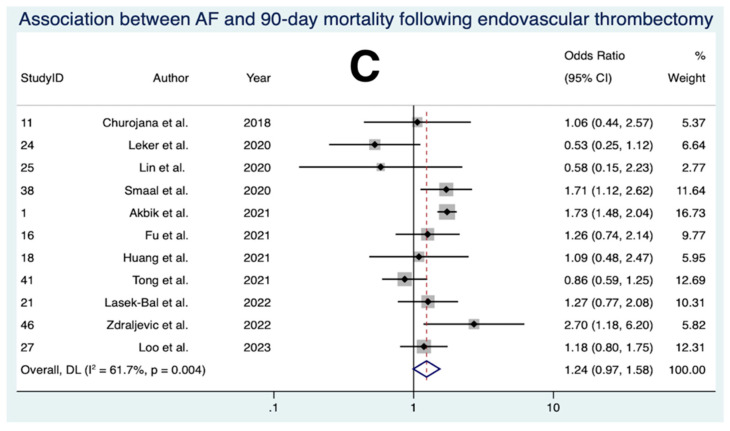
**Forest plots of the association between atrial fibrillation and outcomes following endovascular thrombectomy.** (**A**) association between atrial fibrillation and favourable 90-day functional outcomes. (**B**) association between atrial fibrillation and symptomatic intracerebral haemorrhage. (**C**) association between atrial fibrillation and 90-day mortality. Abbreviations: AF = atrial fibrillation, CI = confidence interval, sICH = symptomatic intracerebral haemorrhage, DL = DerSimonian–Laird.

Eleven studies, comprising a total of 7409 patients, were included in the final meta-analysis for the association between AF and favourable 90-day functional outcomes following EVT (Figure 5) [25,26,27,64,68,69,71,72,73,77,78]. Overall, the meta-analysis revealed no significant association between AF and favourable functional outcomes at 90 days following EVT (OR 0.826 [95% CI 0.651 to 1.049], *p* = 0.117). Substantial heterogeneity was found between the studies (I^2^ = 74.0%, [95% CI < 0.1% to 89.2%], *p* < 0.001). Visual inspection of the funnel plot (Supplemental Figure S1) and the output from Egger’s test (Supplemental Table S4) did not provide evidence of significant publication bias.

### 3.9. Association between AF and sICH Following EVT

Eleven studies, comprising a total of 6981 patients, were included in the final meta-analysis for the association between AF and sICH following EVT (Figure 5) [25,26,27,64,68,69,71,72,73,77,78]. The definition of sICH used in each study is described in Table 3. Overall, the meta-analysis revealed no significant association between AF and sICH following EVT (OR 0.982 [95% CI 0.815 to 1.184], *p* = 0.851). Low heterogeneity was found between the studies (I^2^ < 0.1% [95% CI < 0.1% to 36.4%], *p* = 0.703). Visual inspection of the funnel plot (Supplemental Figure S1) and the output from Egger’s test (Supplemental Table S4) did not provide evidence of significant publication bias.

### 3.10. Association between AF and 90-Day Mortality Following EVT

The final meta-analysis encompassed eleven studies involving a total of 7409 patients and investigated the association between AF and 90-day mortality post EVT (Figure 5) [25,26,27,64,68,69,71,72,73,77,78]. Overall, the meta-analysis revealed no statistically significant association between AF and mortality at 90 days post EVT (OR 1.236 [95% CI 0.969 to 1.578], *p* = 0.088). Notably, substantial heterogeneity existed among the studies (I^2^ = 61.7% [95% CI < 0.1% to 83.5%], *p* = 0.004). Both the funnel plot examination (Supplemental Figure S1) and the output from Egger’s test (Supplemental Table S4) did not provide evidence of significant publication bias.

### 3.11. Association between AF and Favourable 90-Day Functional Outcomes Following BT

Three studies, encompassing a total of 4148 patients, investigated the association between AF and favourable 90-day functional outcomes following BT [28,73,81]. In each of these three studies, AF patients reported a decreased rate of favourable 90-day functional outcomes [28,73,81]. However, a meta-analysis could not be performed due to an insufficient number of available studies.

### 3.12. Association between AF and sICH Following BT

Two studies, comprising a total of 4133 patients, reported on the association between AF and sICH following BT [28,81]. The precise definition of sICH employed in each of these studies is outlined in Table 3. Notably, both studies observed a higher incidence of sICH among patients with AF patients [28,81]. Nonetheless, due to scarcity of available studies, a comprehensive meta-analysis could not be undertaken.

### 3.13. Association between AF and 90-Day Mortality Following BT

Three studies, comprising a combined total of 4246 patients, reported on the association between AF and 90-day mortality following BT [28,73,81]. Across all three studies, individuals with AF consistently demonstrated a heightened 90-day mortality rate [28,73,81]. Unfortunately, a comprehensive meta-analysis could not be performed due to limited availability of studies for inclusion.

## 4. Discussion

Our study demonstrated a high prevalence of AF among patients undergoing reperfusion therapy for AIS. To the best of our knowledge, this is the first meta-analysis that estimates the pooled prevalence of AF specifically in AIS patients receiving reperfusion therapy. Whilst the pooled prevalence of AF was high for each form of reperfusion therapy, the highest prevalence was observed in patients undergoing EVT, followed by BT and IVT. Stratifying by region revealed further variations in prevalence. In the context of IVT, AF was associated with significantly lower odds of favourable functional outcomes and higher odds of sICH and mortality in AIS patients. However, no such associations between AF and these clinical outcomes were identified following EVT.

Whilst no previous meta-analyses have investigated AF prevalence in stroke patients receiving reperfusion therapy, comparisons can be drawn with studies encompassing the broader ischaemic stroke population. Interestingly, our analysis suggests a prevalence estimate towards the higher end when contrasted with nationwide studies that reported AF prevalence ranging from 18.2% to 38.0% [85,86,87,88,89]. However, direct comparisons with nationwide studies are potentially misleading due to global variation in AF prevalence [90,91]. Furthermore, our analysis employed a random effects model to determine pooled prevalence [42], thereby mitigating the impact of outliers and studies with disproportionately large sample sizes. Given the established association between AF and increased stroke severity [92], it is plausible that AF patients might have greater eligibility for therapies like EVT when compared to the general stroke population, considering EVT’s recommendation for patients with baseline NIHSS scores of six or more [17]. Therefore, a prudent future direction is to conduct a meta-analysis on the pooled prevalence of AF in the broader ischaemic stroke population. If the prevalence of AF indeed proves higher among AIS patients receiving reperfusion therapy, this would potentially warrant increased resource allocation towards AF detection and treatment within thrombolysis and thrombectomy centres.

When stratified by region, a consistently higher prevalence of AF was observed in East Asian nations (Figure 3). However, genetic variation is unlikely to explain this alone, as studies from the USA indicate a lower AF prevalence among East Asians compared to Caucasians [93,94,95]. Moreover, the higher prevalence in East Asian nations such as Japan can be attributed to their ageing populations, as the prevalence of AF increases with age, aligning with the higher prevalence seen in the patients undergoing reperfusion therapy [96]. Notably, several studies from less developed nations reported a relatively lower AF prevalence, particularly in the context of IVT [24,48,49,60]. This difference could be influenced by limited access to AF diagnostic technologies in these regions, although conclusive insights cannot be drawn due to the lack of relevant studies [97]. Consequently, future epidemiological AF research should prioritise investigating these developing regions.

While no meta-analyses have directly compared AF prevalence among different forms of reperfusion therapy, the observed variation aligns with the existing literature and clinical guidelines. It is logical that AF prevalence is lowest in IVT and highest in EVT, considering pertinent factors. AF patients often receive oral anticoagulant therapy [45,98] which can influence clotting markers [99,100] and contraindicate IVT due to bleeding risks [17]. EVT is recommended for large vessel occlusion (LVO) strokes [17], which often have a cardioembolic source, frequently AF, explaining the higher AF prevalence in patients receiving EVT [27,101]. This implies that AF suspicion is heightened in EVT-treated patients with unknown stroke aetiology, necessitating adequate cardiac monitoring [101].

Our study reaffirms that AF is associated with worse clinical outcomes following IVT, aligning with existing meta-analyses [34,102]. Two recent studies from 2022 were added to our analysis [53,61]. Some studies were excluded for reasons such as an inability to extract the precise numbers for each outcome [51,63] or the follow-up not being at 90 days [103]. In the broad context, our findings underscore the negative association between AF and clinical outcomes following IVT. It is vital that clinicians are aware of this, as it may influence their management plan and how they communicate the risk of treatments with patients. A pathophysiological hypothesis for this association is that the cardiac emboli induced by AF may exhibit greater resistance to IVT [104] due to the thrombi containing a smaller proportion of fibrin compared to erythrocytes [105]. However, the suggestion that cardiac emboli have a lower fibrin content has been disputed by a recent meta-analysis [106]. Further histopathological studies are necessary for clarity. Higher rates of oral antithrombotic treatment among AF patients in some studies in this meta-analysis [23,53,61] may explain the elevated odds of sICH [34]. AF cohorts also reported consistently higher mean ages and baseline NIHSS scores (Table 1), which are independent predictors of poor prognosis [107,108,109]. There is conflicting evidence regarding how baseline blood pressure at the time of thrombolysis influences outcomes [110,111]. This is particularly relevant in the context of AF, as hypertension is more common in AF patients [112]. Furthermore, alteplase was the thrombolytic drug of choice for all IVT studies in this meta-analysis. With evolving interest in tenecteplase for AIS [113,114,115], future research must explore AF’s role in clinical outcomes following tenecteplase.

In contrast to IVT, our analysis did not find statistically significant associations between AF and clinical outcomes following EVT. This contrasts with a meta-analysis which reported that AF was associated with significantly lower odds of favourable 90-day functional outcomes and higher odds of 90-day mortality following EVT [116]. A major difference between our analyses is that we included two additional studies [25,73]. Both studies reported no significant associations and constituted considerable weights in the meta-analyses (Figure 5). There was an outlier in the analysis for 90-day functional outcomes with a strikingly higher odds ratio [72] (Figure 5). This single-centre study contained a relatively small sample size of 83 patients and the authors acknowledged potential biases arising from the devices used for EVT varying between different clinicians. If this study is omitted from the meta-analysis, the upper limit of the confidence interval for the odds of favourable 90-day functional outcomes falls below one (Supplemental Figure S2), representing a statistically significant association. Overall, our findings suggest that the association between AF and clinical outcomes following EVT is yet to be fully understood. The underlying pathophysiological basis for potential associations also remains unresolved. Whilst non-AF patients with AIS exhibit a higher prevalence of intracranial atherosclerosis, which is associated with a refractory response to EVT [117], those with AF are more likely to have a poor collateral status [118]. Similar to the IVT studies, potential confounding arises from higher rates of comorbidities [116] and higher mean ages and baseline NIHSS scores in AF cohorts (Table 1). The varying rates of prior IVT use among the EVT cohorts could have further skewed results [27,68]. After adjusting for covariates, a meta-analysis of six randomised controlled trials (RCTs) revealed no association between AF and poorer clinical outcomes following EVT [78]. In the context of our study, we reported comorbidity rates (Table 2) and compared mean ages and baseline NIHSS scores between AF and non-AF cohorts (Table 1). However, the limitation lies in our inability to fully adjust for these covariates.

Whilst there was not a sufficient number of studies to facilitate a robust meta-analysis concerning AF and clinical outcomes following BT, the data from individual studies is presented in Table 3. The impact of BT on post-stroke outcomes is an increasingly explored field [29,30,82,83,84,119], with a growing interest in understanding how AF modifies this effect. However, there remains a need for more primary studies that specifically investigate the interaction between AF and the effects of BT. Some studies have delved into potential differences in clinical outcomes between AF patients treated with BT and those receiving EVT alone [28,73,74,81,120,121]. It has been postulated that the additional IVT could be detrimental in AF patients, as it may increase the odds of sICH without enhancing functional outcomes [28]. However, other studies present contrasting findings [73,74,121], warranting a meta-analysis that pools this data for a more comprehensive analysis. This represents a crucial avenue for future research, as it strives to ascertain whether BT offers a net advantage for AF patients or if treatment might be judiciously withheld under certain circumstances.

## 5. Limitations

This study had several limitations. Firstly, the strict inclusion criteria caused numerous studies to be omitted, even though including these studies would have enabled more comprehensive meta-analyses for AF prevalence. Most prominently, the age requirement of over 18 years with no upper limit resulted in studies with a pre-defined age range (e.g., 18 to 80 years old) being excluded. Whilst the Modified Jadad Analysis (Supplemental Table S3) suggested the RCTs were generally of higher study quality, AF was usually not a focus of these trials. Thus, the data comparing clinical outcomes between AF and non-AF patients was primarily derived from cohort studies, which lack blinding and placebo controls. A large proportion of these studies were retrospective, which are further prone to selection bias and recall bias [122]. To account for this, subgroup analyses were conducted that segregated the data from prospective and retrospective studies (Supplemental Figures S3 and S4).

There was sizeable heterogeneity within numerous analyses in this study. A major contributor to this heterogeneity is AF detection methods varying from solely checking a patient’s previous medical records [25,33], conducting an electrocardiogram (ECG) on admission [68], 24-h ECG monitoring [23,74] or 30-day cardiac monitoring post-discharge [71]. However, most studies did not describe how AF was detected. These variations are likely to distort the reported AF prevalence, as longer-term cardiac monitoring leads to markedly higher detection rates [123,124,125,126]. Additionally, AF is a clinically diverse condition with subtypes such as paroxysmal, persistent, and permanent AF [127,128], which most studies did not differentiate between. Limited reporting on the existing anticoagulant treatment regimen of patients also contributes to variability in the results since this influences both the risk of stroke [129] and the clinical outcomes following reperfusion therapy [130]. Due to the already expansive scope of this study, we were unable to investigate other important clinical outcomes such as haemorrhagic transformation and successful reperfusion rates. AF is also a risk factor for stroke recurrence [131], which was rarely reported upon by the studies within this meta-analysis. Lastly, whilst functional outcomes and mortality data were frequently available at the chosen 90-day timepoint, more studies are needed that investigate how AF impacts clinical outcomes at follow-up periods of one year and beyond.

## 6. Conclusions

In conclusion, our study is the first meta-analysis to our knowledge that estimates the pooled prevalence of AF amongst patients receiving reperfusion therapy following AIS. The prevalence of AF was highest in patients receiving EVT, followed by BT and IVT, respectively. AF was associated with significantly lower odds of favourable 90-day functional outcomes and significantly higher odds of sICH and 90-day mortality following IVT. Nevertheless, no significant associations between AF and these clinical outcomes were observed after EVT. While the treatment of AF using IVT and BT was examined, the limited number of available studies prevented a comprehensive meta-analysis for BT. Nevertheless, the findings of this study suggest that the treatment of AF using IVT (as assessed through current meta-analysis) and BT (based on trends from available studies [28,73,81]) is associated with poorer outcomes in contrast to EVT alone. These findings suggest potential benefits for patients diagnosed with AF, encouraging consideration of referral for EVT in AIS management. Importantly, it must be underscored that in cases where EVT is not a feasible option, choosing IVT alone presents as a more favourable alternative than receiving no treatment at all. Awareness of these associations among clinicians is vital for informed risk stratification and effectively communicating anticipated prognoses to patients and their families. Further primary research investigating the impact of AF on clinical outcomes after BT is highly recommended.

## Figures and Tables

**Table 1:** Baseline characteristics of studies included within the meta-analysis.

Study ID	Author	Year	Country	Centres	Study Type	Reperfusion Type	AF (n)	Overall (n)	Age ± SD ^a^			Male (%) ^a^			Baseline NIHSS Score ± SD ^a^		
									AF	Non-AF	Overall	AF	Non-AF	Overall	AF	Non-AF	Overall
1	Akbik et al. [64]	2021	International	15	Retrospective	EVT	1517	4169	76 ± 11	65 ± 15	-	42.2	50.1	-	16 ± 6	15 ± 7	-
2	Akbik et al. [28]	2022	International	22	Retrospective	BT	1036	3140	76 ± 11	65 ± 15	-	46.0	52.0	-	16 ± 6	15 ± 7	-
3	Al-Khaled et al. [46]	2014	Germany	15	Prospective	IVT	387	1007	-	-	71.5 ± 12.2	-	-	49.6	-	-	11.6 ± 5.6
4	Alobaida et al. [33]	2023	International	27	Retrospective	EVT	1718	3106	73.6 ± 12.6	61.1 ± 14.8	-	47.1	57.1	-	-	-	-
4a	Alobaida et al. [33]	2023	International	27	Retrospective	BT	549	889	-	-	-	-	-	-	-	-	-
5	Awadh et al. [47]	2010	Scotland	1	Retrospective	IVT	74	228	76 ± 10	66.4 ± 13.4	-	40.5	59.1	-	13.7 ± 8.3	13.8 ± 9.7	-
6	Bavarsad Shahripour et al. [79]	2022	USA	5	Prospective	BT	17	45	76.4 ± 11.3	65.4 ± 16.5	-	58.8	71.4	-	-	-	-
7	Berkhemer et al. [65]	2015	The Netherlands	16	Prospective RCT	EVT	66	233	-	-	65.4 ± 16.0	-	-	57.9	17.3 ± 5.2	-	17.3 ± 5.2
8	Campbell et al. [80]	2015	Australia and New Zealand	14	Prospective RCT	BT	12	35	-	-	68.6 ± 12.3	-	-	48.6	16.7 ± 5.4	-	16.7 ± 5.4
9	Casetta et al. [66]	2022	Italy	-	Prospective	EVT	1193	3422	-	-	70.6	-	-	47.4	-	-	-
10	Chalos et al. [81]	2019	The Netherlands	-	Prospective	BT	186	1144	76.3 ± 12.0	68 ± 15.6	-	46.8	54.8	-	16.3 ± 6.7	15 ± 5.9	-
11	Churojana et al. [26]	2018	Thailand	1	Retrospective	EVT	50	134	69.2 ± 12.9	60.2 ± 16	-	54.0	60.7	-	17.4 ± 5.5	17.1 ± 6.3	-
11a	Churojana et al. [26]	2018	Thailand	1	Retrospective	BT	10	29	-	-	-	-	-	-	-	-	-
12	Das et al. [48]	2020	India	1	Prospective	IVT	6	60	-	-	63.9	-	-	56.7	-	-	-
13	Dharmasaroja et al. [49]	2012	Thailand	1	Prospective	IVT	46	194	-	-	64 ± 13	-	-	59.8	-	-	17.3 ± 27.6
14	Fischer et al. [30]	2022	Europe and Canada	48	Prospective RCT	BT	22	198	-	-	72.7 ± 11.9	-	-	49.8	-	-	16.3 ± 6.0
15	Frank et al. [50]	2012	International	-	Retrospective	IVT	639	3027	74.2 ± 9.5	65.7 ± 12.5		47.3	58.2	-	-	-	-
16	Fu et al. [27]	2021	Australia	1	Prospective	EVT	171	349	77 ± 9.7	61 ± 31.4		48.5	58.4	-	17.7 ± 8.2	16.3 ± 9.0	-
16a	Fu et al. [27]	2021	Australia	1	Prospective	BT	53	132	-	-	-	-	-	-	-	-	-
17	Goyal et al. [67]	2015	International	22	Prospective RCT	EVT	61	165	-	-	70.7 ± 15.7	-	-	-	-	-	16.3 ± 5.2
18	Huang et al. [68]	2021	China	-	Prospective	EVT	123	245	73.3 ± 9.0	63 ± 12.8	-	43.9	68.9	-	16.3 ± 5.3	14.7 ± 4.5	-
18a	Huang et al. [68]	2021	China	-	Prospective	BT	33	83	-	-	-	-	-	-	-	-	-
19	Kimura et al. [51]	2009	Japan	-	Prospective	IVT	44	85	77.2 ± 9	69.4 ± 12.5	-	61.4	70.7	-	17.3 ± 6.5	12.3 ± 7.5	-
20	Kurmann et al. [52]	2018	Switzerland	4	Prospective	IVT	586	1775	-	-	69.8	-	-	59.2	-	-	11.3
21	Lasek-Bal et al. [69]	2022	Poland	1	Retrospective	EVT	108	417	74.9 ± 9.2	66.8 ± 14.5	-	45.4	54.7	-	14.0 ± 5.4	12.2 ± 6.5	-
22	LeCouffe et al. [82]	2021	Netherlands, Belgium and France	20	Prospective RCT	BT	63	266	-	-	69 ± 11.9	-	-	54.1	-	-	15.3 ± 7.5
23	Lee et al. [70]	2018	Korea	3	Retrospective	EVT	354	720	-	-	67.5	-	-	55.1	-	-	16.3 ± 6.7
24	Leker et al. [71]	2020	Israel	-	Retrospective	EVT	109	230	-	-	69.3 ± 14.7	39.4	56.2	-	-	-	17.1 ± 6.6
24a	Leker et al. [71]	2020	Israel	-	Retrospective	BT	24	56	-	-	-	-	-	-	-	-	-
25	Lin et al. [72]	2020	Taiwan	1	Retrospective	EVT	43	80	72.6 ± 9.5	70.9 ± 17.3	-	46.5	57.5	-	17.2 ± 5.1	17.9 ± 6.2	-
25a	Lin et al. [72]	2020	Taiwan	1	Retrospective	BT	24	39	-	-	-	-	-	-	-	-	-
26	Lin et al. [53]	2022	Taiwan	30	Prospective	IVT	980	2351	71.7 ± 11.9	66.4 ± 13	-	58.2	66.7	-	-	-	-
27a	Loo et al. [73]	2023	Singapore, Germany, Italy, UK, China, Taiwan	8	Retrospective	BT	182	451	73.2 ± 10.3	65.6 ± 14.1	-	40.7	63.2	-	18.3 ± 8.1	15.9 ± 7.6	-
27	Loo et al. [73]	2023	Singapore, Germany, Italy, UK, China, Taiwan	8	Retrospective	EVT	314	705	73.4 ± 10.5	65.3 ± 14.7	-	43.6	61.9	-	18.4 ± 8.3	16.5 ± 8	-
28	Marko et al. [54]	2020	Austria	38	Retrospective	IVT	5452	18,953	-	-	74.9 ± 13.5	-	-	52.8	-	-	9.3 ± 7.4
29	Mehrpour et al. [24]	2019	Iran	1	Retrospective	IVT	24	118	-	-	66.1 ± 13.4	-	-	66.1	-	-	11.1 ± 5.1
30	Mitchell et al. [83]	2022	Australia, China, New Zealand, Vietnam	25	Prospective RCT	BT	34	147	-	-	69.3 ± 14.2	-	-	59.9	-	-	15 ± 7.5
31	Mujanovic et al. [74]	2022	Europe and Canada	8	Retrospective	EVT	1347	2941	77 ± 11.1	69 ± 15.6	-	43.5	54.5	-	15.7 ± 6.7	14.3 ± 7.4	-
32	Nogueira et al. [75]	2015	USA	13	Retrospective	EVT	413	1122	-	-	67 ± 15	-	-	51.9	-	-	16.7 ± 5.2
33	Padjen et al. [55]	2013	France and Serbia	-	Prospective	IVT	155	734	75.3 ± 12.0	64 ± 17.8	-	41.9	55.6	-	13.3 ± 7.5	10.7 ± 7.4	-
34	Sanak et al. [22]	2010	Czech Republic	1	Retrospective	IVT	66	157	68.1 ± 8.2	66.5 ± 13.6	-	57.6	65.9	-	13.3 ± 5.4	11 ± 5.1	-
35	Sandercock et al. [56]	2012	International	156	Prospective RCT	IVT	473	1515	-	-	-	-	-	48.4	-	-	-
36	Seet et al. [23]	2011	USA	1	Retrospective	IVT	76	214	78.9 ± 9.9	71.5 ± 14.8	-	42.1	53.6	-	13 ± 4.5	12 ± 6.0	-
37	Shon et al. [57]	2016	Korea	4	Prospective	IVT	123	318	-	-	-	-	-	60.4	-	-	12.7 ± 6.7
38	Smaal et al. [78] ^b^	2020	International	-	Prospective RCT	EVT	224	667	72.8 ± 10.1	63.1 ± 13.7	-	52.2	51.0	-	17.5 ± 4.8	16.5 ± 5.2	-
39	Sung et al. [58]	2013	Taiwan	-	Retrospective	IVT	72	143	-	-	68.3/64.6	58.3	64.8	-	-	-	-
40	Tong et al. [59]	2014	USA	-	Retrospective	IVT	1489	7193	-	-	-	-	-	49.5	-	-	-
41	Tong et al. [25]	2021	China	111	Prospective	EVT	550	1705	71 ± 10.4	62.3 ± 11.9	-	44.7	75.5	-	18 ± 5.9	15.7 ± 7.4	-
41a	Tong et al. [25]	2021	China	111	Prospective	BT	145	513	-	-	-	-	-	-	-	-	-
42	Vorasoot et al. [60]	2020	Thailand	7	Retrospective	IVT	177	772	-	-	63 ± 13.2	-	-	54.4	-	-	2.5 ± 5.6
43	Wu et al. [61]	2022	China	8	Retrospective	IVT	242	630	-	-	72.4/61.8	50.0	69.6	-	-	-	-
44	Yang et al. [84]	2020	China	41	Prospective RCT	BT	149	329	-	-	68.7 ± 11.2	-	-	55.0	-	-	17.7 ± 6.0
45	Yoshimura et al. [76]	2018	Japan	46	Prospective	EVT	658	1278	-	-	74.7 ± 11.4	-	-	59.2	-	-	18 ± 7.4
46	Zdraljevic et al. [77]	2022	Serbia	1	Prospective	EVT	62	127	73.3 ± 9.5	59.8 ± 13.3	-	43.5	63.1	-	16.7 ± 5.8	15.5 ± 6.2	-
46a	Zdraljevic et al. [77]	2022	Serbia	1	Prospective	BT	11	32	-	-	-	-	-	53.5	-	-	-
47	Zhang et al. [62]	2010	China	-	Retrospective	IVT	22	53	68.3 ± 8.8	60.7 ± 12.3	-	40.9	74.2	-	12 ± 7.1	9.1 ± 7.3	-
48	Zhao et al. [63]	2017	China	-	Retrospective	IVT	30	123	-	-	65.6 ± 11.8	-	-	62.6	-	-	7.7 ± 6
49	Zi et al. [29]	2021	China	33	Prospective RCT	BT	62	118	-	-	69.3 ± 13.5	-	-	55.9	-	-	16.3 ± 5.3

Abbreviations: AF = atrial fibrillation, n = number of patients, SD = standard deviation, NIHSS = National Institutes of Health Stroke Scale, IVT = intravenous thrombolysis, EVT = endovascular thrombectomy, BT = bridging therapy, USA = United States of America, UK = United Kingdom, RCT = randomised controlled trial. ^a^ Where studies reported the statistics for the AF and non-AF group separately, only these were included in the table. Where the mean age or NIHSS score was reported for two subgroups (e.g., mean age of AF patients above and below age 80), a weighted average was manually calculated, ^b^ This study is a pooled analysis of 6 RCTs and was included since it reports on the clinical outcomes of AF patients. However, it was not included in the meta-analyses for prevalence to avoid overlaps since some of its constituent RCTs were already present in the prevalence analyses.

**Table 2:** Rates of comorbidities within cohorts included in the meta-analysis.

Study ID	Author	Year	Diabetes, n (%)	Lipid Disorders, n (%)	Hypertension, n (%)	CAD, n (%)	Heart Failure, n (%) ^d^	Previous Stroke/TIA, n (%)	Smoking, n (%)
1	Akbik et al. [64]	2021	1174 (28.21)	1647 (39.53) ^a^	3112 (74.65)	-	-	383 (12.55) ^d^	-
2	Akbik et al. [28]	2022	784 (25.02)	1255 (39.99) ^a^	2247 (71.56)	-	-	320 (12.97) ^d^	-
3	Al-Khaled et al. [46]	2014	198 (19.66)	477 (47.37) ^b^	795 (78.95)	-	-	197 (19.56) ^d^	-
4	Alobaida et al. [33]	2023	945 (30.42)	1550 (51.39) ^a^	2398 (77.21)	870 (28.01)	906 (29.17)	304 (9.79) ^e^	-
4a	Alobaida et al. [33]	2023	-	-	-	-	-	-	-
5	Awadh et al. [47]	2010	24 (10.53)	48 (21.05) ^a^	151 (66.23)	-	-	45 (19.74) ^f^	56 (24.56) ^i^
6	Bavarsad Shahripour et al. [79]	2022	5 (11.11)	-	28 (62.22)	8 (17.78)	6 (13.33)	-	4 (8.89) ^g^
7	Berkhemer et al. [65]	2015	34 (14.59)	58 (24.89) ^a^	98 (42.06)	-	-	29 (12.45) ^d^	65 (28.89) ^g^
8	Campbell et al. [80]	2015	2 (5.71)	-	21 (60.00)	-	-	-	12 (34.29) ^i^
9	Casetta et al. [66]	2022	519 (15.46)	786 (23.41) ^c^	2099 (62.51)	-	-	-	572 (17.04) ^h^
10	Chalos et al. [81]	2019	197 (17.06)	-	562 (49.08)	-	-	164 (14.21) ^d^	-
11	Churojana et al. [26]	2018	-	-	-	-	-	-	-
11a	Churojana et al. [26]	2018	-	-	-	-	-	-	-
12	Das et al. [48]	2020	27 (45.00)	36 (60.00) ^b^	44 (73.33)	7 (11.67)	-	9 (15.00) ^e^	15 (25.00) ^g^
13	Dharmasaroja et al. [49]	2012	50 (25.77)	61 (31.44) ^a^	116 (59.79)	28 (14.43)	-	28 (14.43) ^d^	-
14	Fischer et al. [30]	2022	-	71 (36.60) ^b^	118 (58.42)	-	-	20 (9.95) ^d^, 14 (7.00) ^e^	-
15	Frank et al. [50]	2012	543 (17.94)	-	1930 (63.76)	-	-	-	-
16	Fu et al. [27]	2021	88 (25.21)	205 (58.74) ^a^	250 (71.63)	-	36 (10.32)	73 (20.92) ^f^	68 (19.48) ^g^
16a	Fu et al. [27]	2021	-	-	-	-	-	-	-
17	Goyal et al. [67]	2015	33 (20.00)	58 (35.15) ^a^	105 (63.64)	40 (24.24)	24 (14.55)	17 (10.30) ^d^	80 (48.48) ^h^
18	Huang et al. [68]	2021	37 (15.10)	9 (3.67) ^a^	140 (57.14)	45 (18.37)	-	52 (21.22) ^d^	71 (28.98) ^i^
18a	Huang et al. [68]	2021	-	-	-	-	-	-	-
19	Kimura et al. [51]	2009	17 (20.00)	19 (22.35) ^a^	49 (57.65)	-	-	-	-
20	Kurmann et al. [52]	2018	309 (16.66)	910 (50.58) ^a^	1272 (68.39)	370 (20.01)	-	-	369 (19.79) ^g^
21	Lasek-Bal et al. [69]	2022	105 (25.18)	168 (40.29) ^c^	315 (75.54)	221 (54.17)	-	-	115 (37.10) ^i^
22	LeCouffe et al. [82]	2021	50 (18.80)	73 (27.44) ^b^	139 (52.45)	-	15 (5.64)	44 (16.54) ^d^	66 (25.38) ^h^
23	Lee et al. [70]	2018	-	-	-	-	-	-	-
24	Leker et al. [71]	2020	73 (31.74)	111 (48.26) ^a^	157 (68.26)	-	-	36 (15.65) ^d^	58 (25.22) ^i^
24a	Leker et al. [71]	2020	-	-	-	-	-	-	-
25	Lin et al. [72]	2020	19 (22.89)	34 (40.96) ^a^	53 (63.86)	17 (20.48)	-	18 (21.69) ^d^	21 (25.30) ^i^
25a	Lin et al. [72]	2020	-	-	-	-	-	-	-
26	Lin et al. [53]	2022	755 (32.20)	829 (35.26) ^a^	1679 (71.60)	323 (13.77)	-	-	-
27a	Loo et al. [73]	2023	131 (29.05)	188 (41.69) ^c^	326 (72.28)	75 (16.63)	-	57 (12.64) ^f^	52 (11.53) ^i^
27	Loo et al. [73]	2023	206 (29.22)	283 (40.14) ^c^	509 (72.20)	125 (17.73)	-	115 (16.31) ^f^	81 (11.49) ^g^
28	Marko et al. [54]	2020	3957 (20.88)	10,055 (53.05) ^b^	14,885 (78.54)	-	-	3265 (17.23) ^d^	3099 (16.35) ^g^
29	Mehrpour et al. [24]	2019	41 (34.75)	28 (23.73) ^c^	82 (69.49)	55 (46.61)	-	25 (21.19) ^d^	25 (21.19) ^i^
30	Mitchell et al. [83]	2022	-	-	89 (60.54)	-	-	18 (12.24) ^f^	-
31	Mujanovic et al. [74]	2022	566 (19.54)	1416 (49.13) ^c^	1997 (68.89)	-	-	344 (13.89) ^d^	702 (24.98) ^i^
32	Nogueira et al. [75]	2015	265 (23.62)	-	773 (68.80)	-	-	-	-
33	Padjen et al. [55]	2013	122 (16.62)	336 (45.78) ^b^	483 (65.80)	-	-	78 (10.63) ^d^	203 (27.66) ^g^
34	Sanak et al. [22]	2010	-	-	-	-	-	-	-
35	Sandercock et al. [56]	2012	-	-	-	-	-	-	-
36	Seet et al. [23]	2011	28 (13.08)	109 (50.93) ^a^	164 (76.64)	79 (36.92)	-	47 (21.96) ^f^	29 (13.55) ^g^
37	Shon et al. [57]	2016	94 (29.56)	106 (33.33) ^c^	230 (72.33)	-	-	54 (16.98) ^d^	77 (24.21) ^g^
38	Smaal et al. [78]	2020	114 (17.09)	-	369 (55.32)	-	-	-	-
39	Sung et al. [58]	2013	48 (33.57)	83 (58.04) ^a^	112 (78.32)	-	-	27 (18.88) ^d^	34 (23.78) ^g^
40	Tong et al. [59]	2014	1793 (24.93)	3020 (41.99) ^c^	5757 (80.04)	-	751 (10.44)	1251 (17.39) ^d^	1537 (21.37) ^i^
41	Tong et al. [25]	2021	324 (19.00)	-	1006 (59.00)	-	-	337 (19.77) ^d^	706 (41.41) ^h^
41a	Tong et al. [25]	2021	-	-	-	-	-	-	-
42	Vorasoot et al. [60]	2020	163 (21.11)	137 (17.75) ^c^	378 (48.96)	52 (6.74)	11 (1.42)	118 (15.28) ^d^, 18 (2.33) ^e^	281 (36.40) ^i^
43	Wu et al. [61]	2022	106 (16.83)	216 (34.29) ^a^	481 (76.35)	34 (5.40)	-	98 (15.56) ^d^	221 (35.08) ^g^
44	Yang et al. [84]	2020	65 (19.76)	14 (4.26) ^b^	201 (61.09)	-	17 (5.17)	47 (14.29) ^d^	68 (20.67) ^i^
45	Yoshimura et al. [76]	2018	236 (18.47)	-	739 (57.82)	-	-	95 (7.43) ^d^	183 (14.32) ^g^
46	Zdraljevic et al. [77]	2022	22 (17.32)	75 (59.06) ^a^	102 (80.31)	-	-	19 (14.96) ^d^	32 (25.20) ^i^
46a	Zdraljevic et al. [77]	2022	-	-	-	-	-	-	-
47	Zhang et al. [62]	2010	5 (9.43)	-	26 (49.06)	-	-	-	-
48	Zhao et al. [63]	2017	26 (21.14)	-	83 (67.48)	14 (11.38)	-	40 (32.52) ^d^	49 (39.84) ^i^
49	Zi et al. [29]	2021	20 (16.95)	22 (18.64) ^a^	74 (62.71)	19 (16.10)	-	19 (16.10) ^d^	29 (24.58) ^h^

Abbreviations: TIA = transient ischaemic attack, CAD = coronary artery disease, n = number of patients. ^a^: described as hyperlipidaemia. ^b^: described as hypercholesterolaemia. ^c^: described as dyslipidaemia. ^d^: prior stroke only. ^e^: prior TIA only. ^f^: both stroke and TIA. ^g^: current smokers only. ^h^: previous smokers as well as current smokers. ^i^: does not specify if smokers are current or previous smokers.

**Table 3:** Clinical outcomes of studies within the meta-analysis.

Study ID	Author	Reperfusion Therapy	Good Functional Outcome at 90 Days		sICH		90-Day Mortality		sICH Definition
			AF, n (%)	No AF, n (%)	AF, n (%)	No AF, n (%)	AF, n (%)	No AF, n (%)	
1	Akbik et al. [64]	EVT	426 (31.14)	1029 (42.28)	89 (7.64)	160 (7.28)	354 (25.88)	408 (16.76)	ECASS II
2	Akbik et al. [28]	BT	295 (33.56)	822 (46.13)	91 (9.15)	140 (7.02)	222 (25.26)	324 (18.18)	ECASS II
3	Al-Khaled et al. [46]	IVT	-	-	29 (7.49)	29 (4.68)	-	-	Any bleeding that was not detected on a previous CT scan and associated with an increase in NIHSS score of ≥4
5	Awadh et al. [47]	IVT	-	-	3 (4.05)	7 (4.55)	-	-	Deterioration in NIHSS score of ≥4 within 72 h, and PH1 or PH2 present on CT
10	Chalos et al. [81]	BT	52 (29.89)	373 (42.78)	11 (5.91)	56 (5.85)	65 (34.95)	204 (21.29)	Heidelberg Bleeding Classification
11	Churojana et al. [26]	EVT	19 (38.00)	32 (38.10)	6 (12.00)	11 (13.10)	10 (20.00)	16 (19.05)	NR
13	Dharmasaroja et al. [49]	IVT	-	-	6 (13.04)	5 (3.42)	-	-	NINDS
15	Frank et al. [50]	IVT	211 (33.02)	1179 (49.37)	17 (2.66)	41 (1.72)	139 (21.75)	325 (13.61)	24-h increase in NIHSS score by ≥4 or any stroke/ICH leading to death
16	Fu et al. [27]	EVT	82 (47.95)	85 (47.75)	2 (1.17)	7 (3.93)	37 (21.64)	32 (17.98)	SITS-MOST
18	Huang et al. [68]	EVT	27 (38.57)	31 (44.29)	9 (12.86)	5 (7.14)	15 (21.43)	14 (20.00)	ICH with a 24-h increase in NIHSS score of ≥4
21	Lasek-Bal et al. [69]	EVT	28 (25.93)	89 (28.80)	7 (6.48)	15 (4.85)	30 (27.78)	72 (23.30)	ECASS II
24	Leker et al. [71]	EVT	27 (24.77)	51 (42.15)	6 (5.50)	5 (4.13)	12 (11.01)	23 (19.01)	ECASS III
25	Lin et al. [72]	EVT	24 (55.81)	7 (17.50)	3 (6.98)	4 (10.00)	4 (9.30)	6 (15.00)	SITS-MOST
26	Lin et al. [53]	IVT	351 (39.35)	574 (47.87)	12 (1.22)	14 (1.02)	93 (10.43)	106 (8.84)	SITS-MOST
27	Loo et al. [73]	EVT	106 (34.30)	146 (38.52)	30 (9.55)	51 (13.04)	59 (19.09)	63 (16.62)	SITS-MOST
27a	Loo et al. [73]	BT	63 (35.00)	118 (45.21)	-	-	34 (18.89)	41 (15.71)	-
29	Mehrpour et al. [24]	IVT	5 (20.83)	55 (58.51)	-	-	-	-	-
33	Padjen et al. [55]	IVT	74 (47.74)	375 (64.77)	-	-	34 (21.94)	52 (8.98)	-
34	Sanak et al. [22]	IVT	33 (50.00)	66 (72.53)	3 (4.55)	0 (0.00)	-	-	ECASS II
36	Seet et al. [23]	IVT	32 (42.11)	77 (55.80)	10 (13.16)	7 (5.07)	-	-	Haemorrhagic transformation associated with an increase in NIHSS score of ≥4
38	Smaal et al. [78]	EVT	95 (42.41)	213 (48.19)	8 (3.57)	20 (4.51)	46 (20.54)	58 (13.12)	Various
39	Sung et al. [58]	IVT	34 (47.22)	24 (33.80)	6 (8.33)	66 (9.86)	4 (5.56)	8 (11.27)	ECASS II
40	Tong et al. [59]	IVT	-	-	98 (6.58)	225 (3.94)	-	-	CT showing intracranial haemorrhage and medical records noting clinical deterioration due to haemorrhage
41	Tong et al. [25]	EVT	160 (41.34)	155 (40.16)	36 (9.45)	35 (9.07)	63 (16.28)	71 (18.39)	Heidelberg Bleeding Classification
43	Wu et al. [61]	IVT	97 (40.08)	279 (71.91)	-	-	39 (16.12)	22 (5.67)	-
46	Zdraljevic et al. [77]	EVT	19 (30.65)	35 (53.85)	5 (8.06)	4 (6.15)	22 (35.48)	11 (16.92)	SITS-MOST
47	Zhang et al. [62]	IVT	9 (40.91)	17 (54.84)	4 (18.18)	2 (6.45)	4 (18.18)	3 (9.68)	ECASS III

Abbreviations: n = number of patients, EVT = endovascular thrombectomy, BT = bridging therapy, IVT = intravenous thrombolysis, AF = atrial fibrillation, sICH = symptomatic intracerebral haemorrhage, ECASS = European Cooperative Acute Stroke Study, NIHSS = National Institutes of Health Stroke Scale, CT = computed tomography, PH1 = parenchymal haematoma type 1, PH2 = parenchymal haematoma type 2, NR = not reported, NINDS = National Institute of Neurological Disorders and Stroke, ICH = intracerebral haemorrhage, SITS-MOST = Safe Implementation of Thrombolysis in Stroke-Monitoring Study.

**Table 4:** Summary effect sizes and heterogeneity from the meta-analysis.

Outcome	Reperfusion Therapy	Effect Measure	Effect Measure (95% CI)	Test of ES = 0	Tests of Overall Effect	Heterogeneity					Heterogeneity Variance Estimates
						Chi-Squared	Cochran’s Q	*p*-Value	H (95% CI)	I^2^ (%) (95% CI)	tau^2^
Prevalence	IVT	Prevalence	0.31 (0.28 to 0.35)	*p* < 0.01 z = 30.02	-	738.91	-	<0.01	-	97.29	0.03
	EVT	Prevalence	0.42 (0.38 to 0.46)	*p* < 0.01 z = 30.94	-	606.22	-	<0.01	-	97.20	0.03
	BT	Prevalence	0.36 (0.28 to 0.43)	*p* < 0.001 z = 14.86	-	630.87	-	<0.01	-	97.31	0.10
90-day mRS 0–2	IVT	OR	0.512 (0.376 to 0.696)	-	*p* < 0.001 z = −4.271	-	43.85	<0.001	2.341 (1.000 to 3.897)	81.8 (<0.1 to 93.4)	0.1457
	EVT	OR	0.826 (0.651 to 1.049)	-	*p* = 0.117 z = −1.568	-	38.44	<0.001	1.961 (1.000 to 3.050)	74.0 (<0.1 to 89.2)	0.1009
sICH	IVT	OR	1.690 (1.400 to 2.039)	-	*p* < 0.001 z = 5.473	-	8.14	0.520	0.951 (1.000 to 1.387)	<0.1 (<0.1 to 48.0)	<0.0001
	EVT	OR	0.982 (0.815 to 1.184)	-	*p* = 0.851 z = −0.188	-	7.23	0.703	0.851 (1.000 to 1.254)	<0.1 (<0.1 to 36.4)	<0.0001
90-day mortality	IVT	OR	1.799 (1.218 to 2.657)	-	*p* = 0.003 z = 2.953	-	19.34	0.002	1.966 (1.000 to 3.432)	74.1 (<0.1 to 91.5)	0.1407
	EVT	OR	1.236 (0.969 to 1.578)	-	*p* = 0.088 z = 1.706	-	26.14	0.004	1.617 (1.000 to 2.465)	61.7 (<0.1 to 83.5)	0.0857

Abbreviations: IVT = intravenous thrombolysis, EVT = endovascular thrombectomy, BT = bridging therapy, mRS = modified Rankin Scale, sICH = symptomatic intracerebral haemorhage, OR = odds ratio, ES = effect size, CI = confidence interval, H = relative excess in Cochran’s Q over its degrees-of-freedom, I^2^ = proportion of total variation in effect estimate due to between-study heterogeneity (based on Q).

## Data Availability

The original contributions presented in the study are included in the article and Online Supplemental Information, and further inquiries can be directed to the corresponding author.

## Supplementary Materials

The following supporting information can be downloaded at https://www.mdpi.com/article/10.3390/neurolint15030065/s1: Search Strategy; Supplemental Table S1. Preferred Reporting Items for Systematic Reviews and Meta-Analyses (PRISMA) 2020 checklist; Supplemental Table S2. Meta-analysis of Observational Studies in Epidemiology (MOOSE) checklist.; Supplemental Table S3. Methodological quality assessment of included studies using the modified Jadad scale and assessment of funding bias.; Supplemental Table S4. Outputs from Egger’s test for publication bias; Supplemental Figure S1. Forest plots of the pooled prevalence of atrial fibrillation in acute ischaemic stroke patients treated with reperfusion therapy, stratified by study type; Supplemental Figure S2. Forest plots of the association between atrial fibrillation and outcomes following intravenous thrombolysis, stratified by study type; Supplemental Figure S3. Forest plots of the association between atrial fibrillation and outcomes following endovascular thrombectomy, stratified by study type; Supplemental Figure S4. Graphs of Egger’s regression tests for the meta-analyses on the association between atrial fibrillation and clinical outcomes following reperfusion therapy; Supplemental Figure S5. Funnel plots of meta-analyses on the association between atrial fibrillation and clinical outcomes following reperfusion therapy; Supplemental Figure S6. Sensitivity analyses for meta-analyses on the association between atrial fibrillation and clinical outcomes following reperfusion therapy.

## References

[1] Dong X.J., Wang B.B., Hou F.F., Jiao Y., Li H.W., Lv S.P., Li F.H. (2023). Global burden of atrial fibrillation/atrial flutter and its attributable risk factors from 1990 to 2019. Europace.

[2] Li H., Song X., Liang Y., Bai X., Liu-Huo W.S., Tang C., Chen W., Zhao L. (2022). Global, regional, and national burden of disease study of atrial fibrillation/flutter, 1990–2019: Results from a global burden of disease study, 2019. BMC Public Health.

[3] Lippi G., Sanchis-Gomar F., Cervellin G. (2021). Global epidemiology of atrial fibrillation: An increasing epidemic and public health challenge. Int. J. Stroke.

[4] Islam S.M.S., Maddison R., Uddin R., Ball K., Livingstone K.M., Khan A., Salmon J., Ackerman I.N., Adair T., Adegboye O.A. (2023). The burden and trend of diseases and their risk factors in Australia, 1990–2019: A systematic analysis for the Global Burden of Disease Study 2019. Lancet Public Health.

[5] Jiang S., Seslar S.P., Sloan L.A., Hansen R.N. (2022). Health care resource utilization and costs associated with atrial fibrillation and rural-urban disparities. J. Manag. Care Spec. Pharm..

[6] Lee E., Choi E.K., Han K.D., Lee H., Choe W.S., Lee S.R., Cha M.J., Lim W.H., Kim Y.J., Oh S. (2018). Mortality and causes of death in patients with atrial fibrillation: A nationwide population-based study. PLoS ONE.

[7] Kamel H., Okin P.M., Elkind M.S., Iadecola C. (2016). Atrial Fibrillation and Mechanisms of Stroke: Time for a New Model. Stroke.

[8] Hald E.M., Rinde L.B., Lochen M.L., Mathiesen E.B., Wilsgaard T., Njolstad I., Braekkan S.K., Hansen J.B. (2018). Atrial Fibrillation and Cause-Specific Risks of Pulmonary Embolism and Ischemic Stroke. J. Am. Heart Assoc..

[9] Sposato L.A., Cipriano L.E., Saposnik G., Ruiz Vargas E., Riccio P.M., Hachinski V. (2015). Diagnosis of atrial fibrillation after stroke and transient ischaemic attack: A systematic review and meta-analysis. Lancet Neurol..

[10] Gladstone D.J., Bui E., Fang J., Laupacis A., Lindsay M.P., Tu J.V., Silver F.L., Kapral M.K. (2009). Potentially preventable strokes in high-risk patients with atrial fibrillation who are not adequately anticoagulated. Stroke.

[11] Carnicelli A.P., Hong H., Connolly S.J., Eikelboom J., Giugliano R.P., Morrow D.A., Patel M.R., Wallentin L., Alexander J.H., Cecilia Bahit M. (2022). Direct Oral Anticoagulants versus Warfarin in Patients with Atrial Fibrillation: Patient-Level Network Meta-Analyses of Randomized Clinical Trials with Interaction Testing by Age and Sex. Circulation.

[12] Hart R.G., Pearce L.A., Aguilar M.I. (2007). Meta-analysis: Antithrombotic therapy to prevent stroke in patients who have nonvalvular atrial fibrillation. Ann. Intern. Med..

[13] Tokunaga K., Koga M., Itabashi R., Yamagami H., Todo K., Yoshimura S., Kimura K., Sato S., Terasaki T., Inoue M. (2019). Prior Anticoagulation and Short- or Long-Term Clinical Outcomes in Ischemic Stroke or Transient Ischemic Attack Patients with Nonvalvular Atrial Fibrillation. J. Am. Heart Assoc..

[14] Evans A., Perez I., Yu G., Kalra L. (2001). Should stroke subtype influence anticoagulation decisions to prevent recurrence in stroke patients with atrial fibrillation?. Stroke.

[15] Anderson J.L., Horne B.D., Stevens S.M., Grove A.S., Barton S., Nicholas Z.P., Kahn S.F., May H.T., Samuelson K.M., Muhlestein J.B. (2007). Randomized trial of genotype-guided versus standard warfarin dosing in patients initiating oral anticoagulation. Circulation.

[16] Wańkowicz P., Staszewski J., Dębiec A., Nowakowska-Kotas M., Szylińska A., Rotter I. (2021). Ischemic Stroke Risk Factors in Patients with Atrial Fibrillation Treated with New Oral Anticoagulants. J. Clin. Med..

[17] Powers W.J., Rabinstein A.A., Ackerson T., Adeoye O.M., Bambakidis N.C., Becker K., Biller J., Brown M., Demaerschalk B.M., Hoh B. (2019). Guidelines for the Early Management of Patients with Acute Ischemic Stroke: 2019 Update to the 2018 Guidelines for the Early Management of Acute Ischemic Stroke: A Guideline for Healthcare Professionals from the American Heart Association/American Stroke Association. Stroke.

[18] Oliveira A.J.F., Viana S.M.N., Santos A.S. (2022). Mechanical thrombectomy for acute ischemic stroke: Systematic review and meta-analysis. Einstein.

[19] Kimura K., Iguchi Y., Yamashita S., Shibazaki K., Kobayashi K., Inoue T. (2008). Atrial fibrillation as an independent predictor for no early recanalization after IV-t-PA in acute ischemic stroke. J. Neurol. Sci..

[20] Brinjikji W., Madalina Mereuta O., Dai D., Kallmes D.F., Savastano L., Liu Y., Nimjee S.M., Nogueira R.G., Abbasi M., Kadirvel R. (2021). Mechanisms of fibrinolysis resistance and potential targets for thrombolysis in acute ischaemic stroke: Lessons from retrieved stroke emboli. Stroke Vasc. Neurol..

[21] Huu An N., Dang Luu V., Duy Ton M., Anh Tuan T., Quang Anh N., Hoang Kien L., Tat Thien N., Viet Phuong D., Minh Duc N. (2022). Thrombectomy Alone versus Bridging Therapy in Acute Ischemic Stroke: Preliminary Results of an Experimental Trial. Clin. Ter..

[22] Sanak D., Herzig R., Kral M., Bartkova A., Zapletalova J., Hutyra M., Skoloudik D., Vlachova I., Veverka T., Horak D. (2010). Is atrial fibrillation associated with poor outcome after thrombolysis?. J. Neurol..

[23] Seet R.C., Zhang Y., Wijdicks E.F., Rabinstein A.A. (2011). Relationship between chronic atrial fibrillation and worse outcomes in stroke patients after intravenous thrombolysis. Arch. Neurol..

[24] Mehrpour M., Afrakhte M., Shojaei S.F., Sohrabi A., Ashayeri R., Esmaeili S., Bahadori M. (2019). Factors predicting the outcome of intravenous thrombolysis in stroke patients before rt-PA administration. Casp. J. Intern. Med..

[25] Tong X., Li S., Liu W., Ren Z., Liu R., Jia B., Zhang X., Huo X., Luo G., Ma G. (2021). Endovascular treatment for acute ischemic stroke in patients with versus without atrial fibrillation: A matched-control study. BMC Neurol..

[26] Churojana A., Mongkolratnan A., Sangpetngam B., Aurboonyawat T., Chankaew E., Withayasuk P., Songsaeng D., Cognard C. (2018). A Comparison of Mechanical Thrombectomy for Large Vessel Occlusion in Acute Ischemic Stroke between Patients with and without Atrial Fibrillation. Siriraj Med. J..

[27] Fu J., Cappelen-Smith C., Edwards L., Cheung A., Mannin N., Wenderoth J., Parsons M., Cordato D. (2021). Comparison of functional outcomes after endovascular thrombectomy in patients with and without atrial fibrillation. Vessel Plus.

[28] Akbik F., Alawieh A., Dimisko L., Howard B.M., Cawley C.M., Tong F.C., Nahab F., Samuels O.B., Maier I., Feng W. (2022). Bridging thrombolysis in atrial fibrillation stroke is associated with increased hemorrhagic complications without improved outcomes. J. Neurointerv. Surg..

[29] Zi W., Qiu Z., Li F., Sang H., Wu D., Luo W., Liu S., Yuan J., Song J., Shi Z. (2021). Effect of Endovascular Treatment Alone vs Intravenous Alteplase Plus Endovascular Treatment on Functional Independence in Patients with Acute Ischemic Stroke: The DEVT Randomized Clinical Trial. JAMA.

[30] Fischer U., Kaesmacher J., Strbian D., Eker O., Cognard C., Plattner P.S., Butikofer L., Mordasini P., Deppeler S., Pereira V.M. (2022). Thrombectomy alone versus intravenous alteplase plus thrombectomy in patients with stroke: An open-label, blinded-outcome, randomised non-inferiority trial. Lancet.

[31] Chen Y., Huang Q.F., Sheng C.S., Zhang W., Shao S., Wang D., Cheng Y.B., Wang Y., Guo Q.H., Zhang D.Y. (2020). Detection rate and treatment gap for atrial fibrillation identified through screening in community health centers in China (AF-CATCH): A prospective multicenter study. PLoS Med..

[32] Orchard J., Li J., Freedman B., Webster R., Salkeld G., Hespe C., Gallagher R., Patel A., Kamel B., Neubeck L. (2020). Atrial Fibrillation Screen, Management, and Guideline-Recommended Therapy in the Rural Primary Care Setting: A Cross-Sectional Study and Cost-Effectiveness Analysis of eHealth Tools to Support All Stages of Screening. J. Am. Heart Assoc..

[33] Alobaida M., Harrison S.L., Lane D.A., Underhill P., Hill A., Lip G.Y.H. (2023). Outcomes in patients with ischaemic stroke undergoing endovascular thrombectomy: Impact of atrial fibrillation. J. Stroke Cerebrovasc. Dis..

[34] Hu Y., Ji C. (2021). Efficacy and safety of thrombolysis for acute ischemic stroke with atrial fibrillation: A meta-analysis. BMC Neurol..

[35] Vinding N.E., Kristensen S.L., Rorth R., Butt J.H., Ostergaard L., Olesen J.B., Torp-Pedersen C., Gislason G.H., Kober L., Kruuse C. (2022). Ischemic Stroke Severity and Mortality in Patients with and without Atrial Fibrillation. J. Am. Heart Assoc..

[36] Richter R.R., Austin T.M. (2012). Using MeSH (medical subject headings) to enhance PubMed search strategies for evidence-based practice in physical therapy. Phys. Ther..

[37] Nunn A., Bath P.M., Gray L.J. (2016). Analysis of the Modified Rankin Scale in Randomised Controlled Trials of Acute Ischaemic Stroke: A Systematic Review. Stroke Res. Treat..

[38] Gardener H., Romano L.A., Smith E.E., Campo-Bustillo I., Khan Y., Tai S., Riley N., Sacco R.L., Khatri P., Alger H.M. (2022). Functional status at 30 and 90 days after mild ischaemic stroke. Stroke Vasc. Neurol..

[39] Wan X., Wang W., Liu J., Tong T. (2014). Estimating the sample mean and standard deviation from the sample size, median, range and/or interquartile range. BMC Med. Res. Methodol..

[40] Jadad A.R., Moore R.A., Carroll D., Jenkinson C., Reynolds D.J., Gavaghan D.J., McQuay H.J. (1996). Assessing the quality of reports of randomized clinical trials: Is blinding necessary?. Control. Clin. Trials.

[41] Shen Y.W., Zhang X.M., Lv M., Chen L., Qin T.J., Wang F., Yang J., Liu P.J., Yang J. (2015). Utility of gonadotropin-releasing hormone agonists for prevention of chemotherapy-induced ovarian damage in premenopausal women with breast cancer: A systematic review and meta-analysis. OncoTargets Ther..

[42] Nyaga V.N., Arbyn M., Aerts M. (2014). Metaprop: A Stata command to perform meta-analysis of binomial data. Arch. Public Health.

[43] DerSimonian R., Laird N. (2015). Meta-analysis in clinical trials revisited. Contemp. Clin. Trials.

[44] Higgins J.P.T.T.J., Chandler J., Cumpston M., Li T., Page M.J., Welch V.A. (2022). Cochrane Handbook for Systematic Reviews of Interventions.

[45] Ajabnoor A.M., Zghebi S.S., Parisi R., Ashcroft D.M., Rutter M.K., Doran T., Carr M.J., Mamas M.A., Kontopantelis E. (2022). Incidence of nonvalvular atrial fibrillation and oral anticoagulant prescribing in England, 2009 to 2019: A cohort study. PLoS Med..

[46] Al-Khaled M., Matthis C., Eggers J. (2014). Predictors of in-hospital mortality and the risk of symptomatic intracerebral hemorrhage after thrombolytic therapy with recombinant tissue plasminogen activator in acute ischemic stroke. J. Stroke Cerebrovasc. Dis..

[47] Awadh M., MacDougall N., Santosh C., Teasdale E., Baird T., Muir K.W. (2010). Early recurrent ischemic stroke complicating intravenous thrombolysis for stroke: Incidence and association with atrial fibrillation. Stroke.

[48] Das S., Mondal G.P., Bhattacharya R., Ghosh K.C., Das S., Pattem H.K., Paul S.A., Patra C. (2020). Predictors of Postthrombolysis Outcome and Symptomatic Postthrombolysis Hemorrhage following Intravenous Thrombolysis with Alteplase for Acute Ischemic Stroke. J. Neurosci. Rural Pract..

[49] Dharmasaroja P.A., Muengtaweepongsa S., Pattaraarchachai J., Dharmasaroja P. (2012). Intracerebral hemorrhage following intravenous thrombolysis in Thai patients with acute ischemic stroke. J. Clin. Neurosci..

[50] Frank B., Fulton R., Weimar C., Shuaib A., Lees K.R., Collaborators V. (2012). Impact of atrial fibrillation on outcome in thrombolyzed patients with stroke: Evidence from the Virtual International Stroke Trials Archive (VISTA). Stroke.

[51] Kimura K., Iguchi Y., Shibazaki K., Iwanaga T., Yamashita S., Aoki J. (2009). IV t-PA therapy in acute stroke patients with atrial fibrillation. J. Neurol. Sci..

[52] Kurmann R., Engelter S.T., Michel P., Luft A.R., Wegener S., Branscheidt M., Eskioglou E., Sirimarco G., Lyrer P.A., Gensicke H. (2018). Impact of Smoking on Clinical Outcome and Recanalization after Intravenous Thrombolysis for Stroke: Multicenter Cohort Study. Stroke.

[53] Lin S.F., Chen C.F., Hu H.H., Ho B.L., Chen C.H., Chan L., Lin H.J., Sun Y., Lin Y.Y., Chen P.L. (2022). Comparison of Different Dosages of Alteplase in Atrial Fibrillation-Related Acute Ischemic Stroke after Intravenous Thrombolysis: A Nationwide, Multicenter, Prospective Cohort Study in Taiwan. J. Am. Heart Assoc..

[54] Marko M., Posekany A., Szabo S., Scharer S., Kiechl S., Knoflach M., Serles W., Ferrari J., Lang W., Sommer P. (2020). Trends of r-tPA (Recombinant Tissue-Type Plasminogen Activator) Treatment and Treatment-Influencing Factors in Acute Ischemic Stroke. Stroke.

[55] Padjen V., Bodenant M., Jovanovic D.R., Ponchelle-Dequatre N., Novakovic N., Cordonnier C., Beslac-Bumbasirevic L., Leys D. (2013). Outcome of patients with atrial fibrillation after intravenous thrombolysis for cerebral ischaemia. J. Neurol..

[56] Sandercock P., Wardlaw J.M., Lindley R.I., Dennis M., Cohen G., Murray G., Innes K., Venables G., Czlonkowska A., Kobayashi A. (2012). The benefits and harms of intravenous thrombolysis with recombinant tissue plasminogen activator within 6 h of acute ischaemic stroke (the third international stroke trial [IST-3]): A randomised controlled trial. Lancet.

[57] Shon S.H., Heo S.H., Kim B.J., Choi H.Y., Kwon Y., Yi S.H., Lee J.S., Kim Y.S., Kim H.Y., Koh S.H. (2016). Predictors of Hemorrhage Volume after Intravenous Thrombolysis. J. Stroke Cerebrovasc. Dis..

[58] Sung S.F., Chen Y.W., Tseng M.C., Ong C.T., Lin H.J. (2013). Atrial fibrillation predicts good functional outcome following intravenous tissue plasminogen activator in patients with severe stroke. Clin. Neurol. Neurosurg..

[59] Tong X., George M.G., Yang Q., Gillespie C. (2014). Predictors of in-hospital death and symptomatic intracranial hemorrhage in patients with acute ischemic stroke treated with thrombolytic therapy: Paul Coverdell Acute Stroke Registry 2008–2012. Int. J. Stroke.

[60] Vorasoot N., Sothornwit J., Chomjit A., Kasemsap N., Tiamkao S., Sawanyawisuth K., Kongbunkiat K. (2020). Factors associated with poor clinical outcome after intravenous recombinant tissue plasminogen activator (rt-PA) for acute ischemic stroke in Northeastern Thailand. J. Med. Assoc. Thail..

[61] Wu H., Liu Y., Miao G., Ge J., You S., Zhang X., Liu H., Zhou Y., Lu T., Cao Y. (2022). Effect of the interaction between atrial fibrillation and rt-PA dose on the prognosis of acute ischaemic stroke with intravenous thrombolysis. Postgrad. Med. J..

[62] Zhang J.B., Ding Z.Y., Yang Y., Sun W., Hai F., Sui X.N., Li X.Y., Wang H.Z., Wang X.T., Zheng J.L. (2010). Thrombolysis with alteplase for acute ischemic stroke patients with atrial fibrillation. Neurol. Res..

[63] Zhao Q., Shan W., Liu L., Fu X., Liu P., Hu Y. (2017). Predictors of functional outcome and hemorrhagic complications in acute ischemic stroke patients treated with intravenous thrombolysis—A retrospective analysis. Int. J. Clin. Pharmacol. Ther..

[64] Akbik F., Alawieh A., Cawley C.M., Howard B.M., Tong F.C., Nahab F., Saad H., Dimisko L., Mustroph C., Samuels O.B. (2021). Differential effect of mechanical thrombectomy and intravenous thrombolysis in atrial fibrillation associated stroke. J. Neurointerv. Surg..

[65] Berkhemer O.A., Fransen P.S., Beumer D., van den Berg L.A., Lingsma H.F., Yoo A.J., Schonewille W.J., Vos J.A., Nederkoorn P.J., Wermer M.J. (2015). A randomized trial of intraarterial treatment for acute ischemic stroke. N. Engl. J. Med..

[66] Casetta I., Fainardi E., Pracucci G., Saia V., Sallustio F., da Ros V., Nappini S., Nencini P., Bigliardi G., Vinci S. (2022). Sex differences in outcome after thrombectomy for acute ischemic stroke. A propensity score-matched study. Eur. Stroke J..

[67] Goyal M., Demchuk A.M., Menon B.K., Eesa M., Rempel J.L., Thornton J., Roy D., Jovin T.G., Willinsky R.A., Sapkota B.L. (2015). Randomized assessment of rapid endovascular treatment of ischemic stroke. N. Engl. J. Med..

[68] Huang K., Zha M., Gao J., Du J., Liu R., Liu X. (2021). Increased intracranial hemorrhage of mechanical thrombectomy in acute ischemic stroke patients with atrial fibrillation. J. Thromb. Thrombolysis.

[69] Lasek-Bal A., Zak A., Binek L., Student S., Tomalski W., Krzan A., Puz P., Uchwat U. (2022). The effect of atrial fibrillation on the safety and efficacy of mechanical thrombectomy in patients with stroke. Pol. Arch. Intern. Med..

[70] Lee J.S., Lee S.J., Hong J.M., Choi J.W., Hong J.H., Chang H.W., Kim C.H., Kim Y.W., Kang D.H., Kim Y.S. (2018). Temporal Changes in Care Processes and Outcomes for Endovascular Treatment of Acute Ischemic Stroke: Retrospective Registry Data from Three Korean Centers. Neurointervention.

[71] Leker R.R., Farraj A., Sacagiu T., Honig A., ElHasan H.A., Gomori J.M., Cohen J.E. (2020). Atrial Fibrillation Treatment Adequacy and Outcome after Endovascular Thrombectomy. J. Stroke Cerebrovasc. Dis..

[72] Lin C.J., Luo C.B., Chien C., Chang F.C., Lin C.J., Lee I.H., Hsu L.C., Chung C.P., Liu H.Y., Chi N.F. (2020). Better endovascular mechanical thrombectomy outcome in atrial fibrillation patients with acute ischemic stroke: A single-center experience. J. Chin. Med. Assoc..

[73] Loo J.H., Leow A.S., Jing M., Sia C.H., Chan B.P., Seet R.C., Teoh H.L., Meyer L., Fiehler J., Papanagiotou P. (2023). Impact of atrial fibrillation on the treatment effect of bridging thrombolysis in ischemic stroke patients undergoing endovascular thrombectomy: A multicenter international cohort study. J. Neurointerv. Surg..

[74] Mujanovic A., Kurmann C.C., Dobrocky T., Olive-Gadea M., Maegerlein C., Pierot L., Mendes Pereira V., Costalat V., Psychogios M., Michel P. (2022). Bridging intravenous thrombolysis in patients with atrial fibrillation. Front. Neurol..

[75] Nogueira R.G., Gupta R., Jovin T.G., Levy E.I., Liebeskind D.S., Zaidat O.O., Rai A., Hirsch J.A., Hsu D.P., Rymer M.M. (2015). Predictors and clinical relevance of hemorrhagic transformation after endovascular therapy for anterior circulation large vessel occlusion strokes: A multicenter retrospective analysis of 1122 patients. J. Neurointerv. Surg..

[76] Yoshimura S., Sakai N., Uchida K., Yamagami H., Ezura M., Okada Y., Kitagawa K., Kimura K., Sasaki M., Tanahashi N. (2018). Endovascular Therapy in Ischemic Stroke with Acute Large-Vessel Occlusion: Recovery by Endovascular Salvage for Cerebral Ultra-Acute Embolism Japan Registry 2. J. Am. Heart Assoc..

[77] Zdraljevic M., Pekmezovic T., Stanarcevic P., Vukasinovic I., Berisavac I., Ercegovac M., Vitosevic F., Nestorovic D., Cvetic V., Padjen V. (2022). Atrial fibrillation is associated with poor long-term outcome after mechanical thrombectomy for anterior large vessel occlusion stroke. J. Stroke Cerebrovasc. Dis..

[78] Smaal J.A., de Ridder I.R., Heshmatollah A., van Zwam W.H., Dippel D., Majoie C.B., Brown S., Goyal M., Campbell B., Muir K.W. (2020). Effect of atrial fibrillation on endovascular thrombectomy for acute ischemic stroke. A meta-analysis of individual patient data from six randomised trials: Results from the HERMES collaboration. Eur. Stroke J..

[79] Bavarsad Shahripour R., Azarpazhooh D., Shifflett B., Osouli S., Meyer B.C., Meyer D.M. (2022). The Impact of Atrial Fibrillation on the Outcome of Ischemic Stroke Treated with Thrombolysis or Endovascular Therapy. J. Neurol. Res..

[80] Campbell B.C., Mitchell P.J., Kleinig T.J., Dewey H.M., Churilov L., Yassi N., Yan B., Dowling R.J., Parsons M.W., Oxley T.J. (2015). Endovascular therapy for ischemic stroke with perfusion-imaging selection. N. Engl. J. Med..

[81] Chalos V., LeCouffe N.E., Uyttenboogaart M., Lingsma H.F., Mulder M., Venema E., Treurniet K.M., Eshghi O., van der Worp H.B., van der Lugt A. (2019). Endovascular Treatment with or without Prior Intravenous Alteplase for Acute Ischemic Stroke. J. Am. Heart Assoc..

[82] LeCouffe N.E., Kappelhof M., Treurniet K.M., Rinkel L.A., Bruggeman A.E., Berkhemer O.A., Wolff L., van Voorst H., Tolhuisen M.L., Dippel D.W.J. (2021). A Randomized Trial of Intravenous Alteplase before Endovascular Treatment for Stroke. N. Engl. J. Med..

[83] Mitchell P.J., Yan B., Churilov L., Dowling R.J., Bush S.J., Bivard A., Huo X.C., Wang G., Zhang S.Y., Ton M.D. (2022). Endovascular thrombectomy versus standard bridging thrombolytic with endovascular thrombectomy within 4.5 h of stroke onset: An open-label, blinded-endpoint, randomised non-inferiority trial. Lancet.

[84] Yang P., Zhang Y., Zhang L., Zhang Y., Treurniet K.M., Chen W., Peng Y., Han H., Wang J., Wang S. (2020). Endovascular Thrombectomy with or without Intravenous Alteplase in Acute Stroke. N. Engl. J. Med..

[85] Bjorck S., Palaszewski B., Friberg L., Bergfeldt L. (2013). Atrial fibrillation, stroke risk, and warfarin therapy revisited: A population-based study. Stroke.

[86] Otite F.O., Khandelwal P., Chaturvedi S., Romano J.G., Sacco R.L., Malik A.M. (2016). Increasing atrial fibrillation prevalence in acute ischemic stroke and TIA. Neurology.

[87] Alkhouli M., Alqahtani F., Aljohani S., Alvi M., Holmes D.R. (2018). Burden of Atrial Fibrillation-Associated Ischemic Stroke in the United States. JACC Clin. Electrophysiol..

[88] Diaz-Guzman J., Freixa-Pamias R., Garcia-Alegria J., Perez Cabeza A.I., Roldan-Rabadan I., Antolin-Fontes B., Rebollo P., Llorac A., Genis-Girones M., Escobar-Cervantes C. (2022). Epidemiology of atrial fibrillation-related ischemic stroke and its association with DOAC uptake in Spain: First national population-based study 2005 to 2018. Rev. Esp. Cardiol. (Engl. Ed.).

[89] Ding M., Ebeling M., Ziegler L., Wennberg A., Modig K. (2023). Time trends in atrial fibrillation-related stroke during 2001–2020 in Sweden: A nationwide, observational study. Lancet Reg. Health Eur..

[90] Dai H., Zhang Q., Much A.A., Maor E., Segev A., Beinart R., Adawi S., Lu Y., Bragazzi N.L., Wu J. (2021). Global, regional, and national prevalence, incidence, mortality, and risk factors for atrial fibrillation, 1990–2017: Results from the Global Burden of Disease Study 2017. Eur. Heart J. Qual. Care Clin. Outcomes.

[91] Joseph P.G., Healey J.S., Raina P., Connolly S.J., Ibrahim Q., Gupta R., Avezum A., Dans A.L., Lopez-Jaramillo P., Yeates K. (2021). Global variations in the prevalence, treatment, and impact of atrial fibrillation in a multi-national cohort of 153 152 middle-aged individuals. Cardiovasc. Res..

[92] Kimura K., Minematsu K., Yamaguchi T., Japan Multicenter Stroke Investigators C. (2005). Atrial fibrillation as a predictive factor for severe stroke and early death in 15,831 patients with acute ischaemic stroke. J. Neurol. Neurosurg. Psychiatry.

[93] Shen A.Y., Contreras R., Sobnosky S., Shah A.I., Ichiuji A.M., Jorgensen M.B., Brar S.S., Chen W. (2010). Racial/ethnic differences in the prevalence of atrial fibrillation among older adults--A cross-sectional study. J. Natl. Med. Assoc..

[94] Dewland T.A., Olgin J.E., Vittinghoff E., Marcus G.M. (2013). Incident atrial fibrillation among Asians, Hispanics, blacks, and whites. Circulation.

[95] Lau C.P., Gbadebo T.D., Connolly S.J., Van Gelder I.C., Capucci A., Gold M.R., Israel C.W., Morillo C.A., Siu C.W., Abe H. (2013). Ethnic differences in atrial fibrillation identified using implanted cardiac devices. J. Cardiovasc. Electrophysiol..

[96] Koga M. (2018). More Benefits From Endovascular Thrombectomy in Patients with Atrial Fibrillation?. Circ. J..

[97] Al-Shamkhani W., Ayetey H., Lip G.Y.H. (2018). Atrial fibrillation in the Middle East: Unmapped, underdiagnosed, undertreated. Expert Rev. Cardiovasc. Ther..

[98] Navar A.M., Kolkailah A.A., Overton R., Shah N.P., Rousseau J.F., Flaker G.C., Pignone M.P., Peterson E.D. (2022). Trends in Oral Anticoagulant Use among 436 864 Patients with Atrial Fibrillation in Community Practice, 2011 to 2020. J. Am. Heart Assoc..

[99] Nelson W.W., Wang L., Baser O., Damaraju C.V., Schein J.R. (2015). Out-of-range INR values and outcomes among new warfarin patients with non-valvular atrial fibrillation. Int. J. Clin. Pharm..

[100] Cho H.J., Kang Y.J., Sung S.M., Ahn S.H., Jung Y.H., Lee K.Y., Seo J.H., Han S.W., Park J.H., Choi H.Y. (2020). Effects of dabigatran and rivaroxaban on stroke severity according to the results of routine coagulation tests. PLoS ONE.

[101] Seachrist E.J., Petrone A., Nevin C., Ranasinghe T., Jacob S., Ferari C., Adcock A. (2023). Incidence of Atrial Fibrillation in Large Vessel Occlusion and Large Embolic Stroke of Undetermined Source. Cureus.

[102] Yue R., Li D., Yu J., Li S., Ma Y., Huang S., Zeng Z., Zeng R., Sun X. (2016). Atrial Fibrillation is Associated with Poor Outcomes in Thrombolyzed Patients with Acute Ischemic Stroke: A Systematic Review and Meta-Analysis. Medicine.

[103] Saposnik G., Gladstone D., Raptis R., Zhou L., Hart R.G., Investigators of the Registry of the Canadian Stroke Network, the Stroke Outcomes Research Canada (SORCan) Working Group (2013). Atrial fibrillation in ischemic stroke: Predicting response to thrombolysis and clinical outcomes. Stroke.

[104] Findler M., Molad J., Bornstein N.M., Auriel E. (2017). Worse Outcome in Patients with Acute Stroke and Atrial Fibrillation Following Thrombolysis. Isr. Med. Assoc. J..

[105] Kim S.K., Yoon W., Kim T.S., Kim H.S., Heo T.W., Park M.S. (2015). Histologic Analysis of Retrieved Clots in Acute Ischemic Stroke: Correlation with Stroke Etiology and Gradient-Echo MRI. AJNR Am. J. Neuroradiol..

[106] Huang J., Killingsworth M.C., Bhaskar S.M.M. (2022). Is Composition of Brain Clot Retrieved by Mechanical Thrombectomy Associated with Stroke Aetiology and Clinical Outcomes in Acute Ischemic Stroke? A Systematic Review and Meta-Analysis. Neurol. Int..

[107] Mohammaden M.H., Stapleton C.J., Brunozzi D., Hussein A.E., Khedr E.M., Atwal G., Alaraj A. (2020). Predictors of Poor Outcome Despite Successful Mechanical Thrombectomy of Anterior Circulation Large Vessel Occlusions within 6 h of Symptom Onset. Front. Neurol..

[108] Jiao J., Liu S., Cui C., Cao Y., Jia Z., Liu H., Wang C., Hang Y., Ni H., Chen M. (2022). Endovascular thrombectomy for acute ischemic stroke in elderly patients with atrial fibrillation. BMC Neurol..

[109] Shao R., Wang Z., Shi H., Li Y., Zhuang Y., Xu J., Xu M. (2022). Stroke severity modified the effect of chronic atrial fibrillation on the outcome of thrombolytic therapy. Medicine.

[110] Lei Z., Li S., Hu S., Ren L. (2020). Effects of Baseline Systolic Blood Pressure on Outcome in Ischemic Stroke Patients with Intravenous Thrombolysis Therapy: A Systematic Review and Meta-Analysis. Neurologist.

[111] Xiaoxi Z., Xuan Z., Lei Z., Zifu L., Pengfei X., Hongjian S., Yongxin Z., Weilong H., Yihan Z., Dongwei D. (2022). Baseline blood pressure does not modify the effect of intravenous thrombolysis in successfully revascularized patients. Front. Neurol..

[112] Aune D., Mahamat-Saleh Y., Kobeissi E., Feng T., Heath A.K., Janszky I. (2023). Blood pressure, hypertension and the risk of atrial fibrillation: A systematic review and meta-analysis of cohort studies. Eur. J. Epidemiol..

[113] Bivard A., Zhao H., Churilov L., Campbell B.C.V., Coote S., Yassi N., Yan B., Valente M., Sharobeam A., Balabanski A.H. (2022). Comparison of tenecteplase with alteplase for the early treatment of ischaemic stroke in the Melbourne Mobile Stroke Unit (TASTE-A): A phase 2, randomised, open-label trial. Lancet Neurol..

[114] Kvistad C.E., Naess H., Helleberg B.H., Idicula T., Hagberg G., Nordby L.M., Jenssen K.N., Tobro H., Rorholt D.M., Kaur K. (2022). Tenecteplase versus alteplase for the management of acute ischaemic stroke in Norway (NOR-TEST 2, part A): A phase 3, randomised, open-label, blinded endpoint, non-inferiority trial. Lancet Neurol..

[115] Wang Y., Li S., Pan Y., Li H., Parsons M.W., Campbell B.C.V., Schwamm L.H., Fisher M., Che F., Dai H. (2023). Tenecteplase versus alteplase in acute ischaemic cerebrovascular events (TRACE-2): A phase 3, multicentre, open-label, randomised controlled, non-inferiority trial. Lancet.

[116] Kobeissi H., Ghozy S., Seymour T., Gupta R., Bilgin C., Kadirvel R., Rabinstein A.A., Kallmes D.F. (2023). Outcomes of Patients with Atrial Fibrillation Following Thrombectomy for Stroke: A Systematic Review and Meta-analysis. JAMA Netw. Open.

[117] Baek J.H., Kim B.M., Heo J.H., Kim D.J., Nam H.S., Kim Y.D. (2018). Outcomes of Endovascular Treatment for Acute Intracranial Atherosclerosis-Related Large Vessel Occlusion. Stroke.

[118] Guglielmi V., LeCouffe N.E., Zinkstok S.M., Compagne K.C.J., Eker R., Treurniet K.M., Tolhuisen M.L., van der Worp H.B., Jansen I.G.H., van Oostenbrugge R.J. (2019). Collateral Circulation and Outcome in Atherosclerotic versus Cardioembolic Cerebral Large Vessel Occlusion. Stroke.

[119] Suzuki K., Matsumaru Y., Takeuchi M., Morimoto M., Kanazawa R., Takayama Y., Kamiya Y., Shigeta K., Okubo S., Hayakawa M. (2021). Effect of Mechanical Thrombectomy without vs With Intravenous Thrombolysis on Functional Outcome Among Patients with Acute Ischemic Stroke: The SKIP Randomized Clinical Trial. JAMA.

[120] Yaghi S., Mistry E., de Havenon A., Leon Guerrero C.R., Nouh A., Liberman A.L., Giles J., Liu A., Nagy M., Kaushal A. (2021). Effect of Alteplase Use on Outcomes in Patients with Atrial Fibrillation: Analysis of the Initiation of Anticoagulation after Cardioembolic Stroke Study. J. Am. Heart Assoc..

[121] Lin L., Blair C., Fu J., Cordato D., Cappelen-Smith C., Cheung A., Manning N.W., Wenderoth J., Chen C., Bivard A. (2023). Prior anticoagulation and bridging thrombolysis improve outcomes in patients with atrial fibrillation undergoing endovascular thrombectomy for anterior circulation stroke. J. Neurointerv. Surg..

[122] Talari K., Goyal M. (2020). Retrospective studies—Utility and caveats. J. R. Coll. Physicians Edinb..

[123] Dahal K., Chapagain B., Maharjan R., Farah H.W., Nazeer A., Lootens R.J., Rosenfeld A. (2016). Prolonged Cardiac Monitoring to Detect Atrial Fibrillation after Cryptogenic Stroke or Transient Ischemic Attack: A Meta-Analysis of Randomized Controlled Trials. Ann. Noninvasive Electrocardiol..

[124] Buck B.H., Hill M.D., Quinn F.R., Butcher K.S., Menon B.K., Gulamhusein S., Siddiqui M., Coutts S.B., Jeerakathil T., Smith E.E. (2021). Effect of Implantable vs Prolonged External Electrocardiographic Monitoring on Atrial Fibrillation Detection in Patients with Ischemic Stroke: The PER DIEM Randomized Clinical Trial. JAMA.

[125] Liu C.M., Chang S.L., Yeh Y.H., Chung F.P., Hu Y.F., Chou C.C., Hung K.C., Chang P.C., Liao J.N., Chan Y.H. (2021). Enhanced detection of cardiac arrhythmias utilizing 14-day continuous ECG patch monitoring. Int. J. Cardiol..

[126] Jiang H., Tan S.Y., Wang J.K., Li J., Tu T.M., Tan V.H., Yeo C. (2022). A meta-analysis of extended ECG monitoring in detection of atrial fibrillation in patients with cryptogenic stroke. Open Heart.

[127] Almeida E.D., Guimaraes R.B., Stephan L.S., Medeiros A.K., Foltz K., Santanna R.T., Pires L.M., Kruse M.L., Lima G.G., Leiria T.L. (2015). Clinical Differences between Subtypes of Atrial Fibrillation and Flutter: Cross-Sectional Registry of 407 Patients. Arq. Bras. Cardiol..

[128] Atar D., Berge E., Le Heuzey J.Y., Virdone S., Camm A.J., Steffel J., Gibbs H., Goldhaber S.Z., Goto S., Kayani G. (2020). The association between patterns of atrial fibrillation, anticoagulation, and cardiovascular events. Europace.

[129] Holthuis E., Smits E., Spentzouris G., Beier D., Enders D., Gini R., Bartolini C., Mazzaglia G., Penning-van Beest F., Herings R. (2022). Increased Risk of Stroke Due to Non-adherence and Non-persistence with Direct Oral Anticoagulants (DOACs): Real-World Analyses Using a Nested Case-Control Study from The Netherlands, Italy and Germany. Drugs Real World Outcomes.

[130] Chen J.H., Hong C.T., Chung C.C., Kuan Y.C., Chan L. (2022). Safety and efficacy of endovascular thrombectomy in acute ischemic stroke treated with anticoagulants: A systematic review and meta-analysis. Thromb. J..

[131] Zheng S., Yao B. (2019). Impact of risk factors for recurrence after the first ischemic stroke in adults: A systematic review and meta-analysis. J. Clin. Neurosci..

