# Supplementary material for: Atrial Fibrillation and Reperfusion Therapy in Acute Ischaemic Stroke Patients: Prevalence and Outcomes—A Comprehensive Systematic Review and Meta-Analysis
Source: Neurol Int. 2023 Aug 25;15(3):1014–43. doi: 10.3390/neurolint15030065 (PMC10537209; doi:10.3390/neurolint15030065)
Supplement: Supplementary file 1 [file neurolint-15-00065-s001.zip › neurolint-2584073-supplementary.pdf]

Supplemental Information

Atrial Fibrillation and Reperfusion Therapy in Acute Ischaemic Stroke Patients: Prevalence and Outcomes—A Comprehensive Systematic Review and Meta-Analysis

Jay Patel [1,2,3] and Sonu M. M. Bhaskar [1,3,4,5,*,†]

1 Global Health Neurology Lab., Sydney, NSW 2150, Australia; jay.patel1@student.unsw.edu.au
2 South Western Sydney Clinical Campuses, University of New South Wales (UNSW) Medicine and Health, UNSW Sydney, Sydney, NSW 2170, Australia
3 Neurovascular Imaging Laboratory, Clinical Sciences Stream, Ingham Institute for Applied Medical Research, Sydney, NSW 2170, Australia
4 NSW Brain Clot Bank, NSW Health Pathology, Sydney, NSW 2170, Australia
5 Department of Neurology & Neurophysiology, Liverpool Hospital & South Western Sydney Local Health District (SWSLHD), Sydney, NSW 2170, Australia
* Correspondence: sonu.bhaskar@globalhealthneurolab.org or bhaskar.sonu@ncvc.go.jp; Tel.: +81-90-9274-1265
† Current address: National Cerebral and Cardiovascular Center (NCVC), Department of Neurology, 6-1 Kishibeshimmachi, Suita 564-8565, Osaka, Japan.

TABLE OF CONTENTS

1) Search Strategy

2) Supplemental Tables

2.1. Supplemental Table S1. Preferred Reporting Items for Systematic Reviews and Meta-Analyses (PRISMA) 2020 checklist.

2.2. Supplemental Table S2. Meta-analysis of Observational Studies in Epidemiology (MOOSE) checklist.

2.3. Supplemental Table S3. Methodological quality assessment of included studies using the modified Jadad scale and assessment of funding bias.

2.4. Supplemental Table S4. Outputs from Egger's test for publication bias.

3) Supplemental Figures

3.1. Supplemental Figure S1. Forest plots of the pooled prevalence of atrial fibrillation in acute ischaemic stroke patients treated with reperfusion therapy, stratified by study type.

3.2. Supplemental Figure S2. Forest plots of the association between atrial fibrillation and outcomes following intravenous thrombolysis, stratified by study type.

3.3. Supplemental Figure S3. Forest plots of the association between atrial fibrillation and outcomes following endovascular thrombectomy, stratified by study type.
3.4. Supplemental Figure S4. Graphs of Egger's regression tests for the meta-analyses on the association between atrial fibrillation and clinical outcomes following reperfusion therapy.
3.5. Supplemental Figure S5. Funnel plots of meta-analyses on the association between atrial fibrillation and clinical outcomes following reperfusion therapy.
3.6. Supplemental Figure S6. Sensitivity analyses for meta-analyses on the association between atrial fibrillation and clinical outcomes following reperfusion therapy.

1. Search strategy

PubMed

("atrial fibrillation"[MeSH Terms] OR "atrial fibrillation"[Title/Abstract] OR "AF"[Title/Abstract] OR "AFib"[Title/Abstract]) AND ("ischemic stroke"[MeSH Terms] OR "cerebrovascular ischemia"[MeSH Terms] OR "brain ischemia"[MeSH Terms] OR "stroke, acute"[MeSH Terms])

Filters applied: Clinical Study, Clinical Trial, Clinical Trial Protocol, Clinical Trial, Phase I, Clinical Trial, Phase II, Clinical Trial, Phase III, Clinical Trial, Phase IV, Comparative Study, Dataset, Evaluation Study, Meta-Analysis, Multicenter Study, Observational Study, Randomized Controlled Trial, Review, Systematic Review, Validation Study, Humans, English, Adult: 19+ years.

Embase

Multi-field search:
"atrial fibrillation" OR "AF" OR "AFib" [Abstract]
AND
"ischemic stroke" OR "ischaemic stroke" OR "brain ischemia" OR "brain ischaemia" OR "cerebrovascular ischemia" OR "cerebrovascular ischaemia" [All fields]
AND
“reperfusion therapy" OR "thrombolysis" OR "IVT" OR "tPA" OR "tissue plasminogen activator" OR "thrombectomy" OR "clot retrieval" OR "EVT" OR "bridging thrombolysis" OR "bridging intravenous thrombolysis" OR "bridging reperfusion therapy" [All fields]

limit 1 to (human and (clinical trial or randomized controlled trial or controlled clinical trial or multicenter study or phase 1 clinical trial or phase 2 clinical trial or phase 3 clinical trial or phase 4 clinical trial)): 241 results.

+

limit 1 to (human and (meta analysis or "systematic review")): 74 results

Cochrane

Title Abstract Keyword: “atrial fibrillation" OR "AF" OR "AFib"

Title Abstract Keyword: "ischemic stroke" OR "cerebrovascular ischemia" OR "brain ischemia" OR "stroke, acute" (Word Variations have been searched)

(("atrial fibrillation":ti,ab,kw OR "AF":ti,ab,kw OR "AFib":ti,ab,kw) AND ("ischemic stroke":ti,ab,kw OR "cerebrovascular ischemia":ti,ab,kw OR "brain ischemia":ti,ab,kw OR "stroke, acute":ti,ab,kw))

2. List of Supplemental Tables

2.1. Supplemental Table S1. Preferred Reporting Items for Systematic Reviews and Meta-Analyses (PRISMA) 2020 checklist.
2.2. Supplemental Table S2. Meta-analysis of Observational Studies in Epidemiology (MOOSE) checklist.
2.3. Supplemental Table S3. Methodological quality assessment of included studies using the modified Jadad scale and assessment of funding bias.
2.4. Supplemental Table S4. Outputs from Egger’s test for publication bias.

2.1. Supplemental Table S1. Preferred Reporting Items for Systematic Reviews and Meta-Analyses (PRISMA) 2020 checklist.

Section and Topic	Item #	Checklist item	Location where item is reported
**TITLE**			
Title	1	Identify the report as a systematic review.	1
**ABSTRACT**			
Abstract	2	See the PRISMA 2020 for Abstracts checklist.	2
**INTRODUCTION**			
Rationale	3	Describe the rationale for the review in the context of existing knowledge.	3
Objectives	4	Provide an explicit statement of the objective(s) or question(s) the review addresses.	3-4
**METHODS**			
Eligibility criteria	5	Specify the inclusion and exclusion criteria for the review and how studies were grouped for the syntheses.	4
Information sources	6	Specify all databases, registers, websites, organisations, reference lists and other sources searched or consulted to identify studies. Specify the date when each source was last searched or consulted.	4
Search strategy	7	Present the full search strategies for all databases, registers, and websites, including any filters and limits used.	4, Supplemental Information
Selection process	8	Specify the methods used to decide whether a study met the inclusion criteria of the review, including how many reviewers screened each record and each report retrieved, whether they worked independently, and if applicable, details of automation tools used in the process.	4-5
Data collection process	9	Specify the methods used to collect data from reports, including how many reviewers collected data from each report, whether they worked independently, any processes for obtaining or confirming data from study investigators, and if applicable, details of automation tools used in the process.	5

Section and Topic	Item #	Checklist item	Location where item is reported
Data items	10a	List and define all outcomes for which data were sought. Specify whether all results that were compatible with each outcome domain in each study were sought (e.g., for all measures, time points, analyses), and if not, the methods used to decide which results to collect.	5
	10b	List and define all other variables for which data were sought (e.g., participant and intervention characteristics, funding sources). Describe any assumptions made about any missing or unclear information.	5
Study risk of bias assessment	11	Specify the methods used to assess risk of bias in the included studies, including details of the tool(s) used, how many reviewers assessed each study and whether they worked independently, and if applicable, details of automation tools used in the process.	5, Supplemental Information
Effect measures	12	Specify for each outcome the effect measure(s) (e.g. risk ratio, mean difference) used in the synthesis or presentation of results.	5
Synthesis methods	13a	Describe the processes used to decide which studies were eligible for each synthesis (e.g., tabulating the study intervention characteristics and comparing against the planned groups for each synthesis (item #5)).	4-5
	13b	Describe any methods required to prepare the data for presentation or synthesis, such as handling of missing summary statistics, or data conversions.	5
	13c	Describe any methods used to tabulate or visually display results of individual studies and syntheses.	5
	13d	Describe any methods used to synthesize results and provide a rationale for the choice(s). If meta-analysis was performed, describe the model(s), method(s) to identify the presence and extent of statistical heterogeneity, and software package(s) used.	5-6
	13e	Describe any methods used to explore possible causes of heterogeneity among study results (e.g., subgroup	6

Section and Topic	Item #	Checklist item	Location where item is reported
		analysis, meta-regression).	
	13f	Describe any sensitivity analyses conducted to assess robustness of the synthesized results.	6
Reporting bias assessment	14	Describe any methods used to assess risk of bias due to missing results in a synthesis (arising from reporting biases).	5-6
Certainty assessment	15	Describe any methods used to assess certainty (or confidence) in the body of evidence for an outcome.	5
**RESULTS**			
Study selection	16a	Describe the results of the search and selection process, from the number of records identified in the search to the number of studies included in the review, ideally using a flow diagram.	6, 38 (PRISMA flowchart)
	16b	Cite studies that might appear to meet the inclusion criteria, but which were excluded, and explain why they were excluded.	-
Study characteristics	17	Cite each included study and present its characteristics.	27-30
Risk of bias in studies	18	Present assessments of risk of bias for each included study.	Supplemental Information
Results of individual studies	19	For all outcomes, present, for each study: (a) summary statistics for each group (where appropriate) and (b) an effect estimate and its precision (e.g. confidence/credible interval), ideally using structured tables or plots.	34-35
Results of syntheses	20a	For each synthesis, briefly summarise the characteristics and risk of bias among contributing studies.	Supplemental Information
	20b	Present results of all statistical syntheses conducted. If meta-analysis was done, present for each the summary estimate and its precision (e.g., confidence/credible interval) and measures of statistical heterogeneity. If comparing groups, describe the direction of the effect.	36
	20c	Present results of all investigations of possible causes of heterogeneity among study results.	-

Section and Topic	Item #	Checklist item	Location where item is reported
	20d	Present results of all sensitivity analyses conducted to assess the robustness of the synthesized results.	Supplemental Information
Reporting biases	21	Present assessments of risk of bias due to missing results (arising from reporting biases) for each synthesis assessed.	Supplemental Information
Certainty of evidence	22	Present assessments of certainty (or confidence) in the body of evidence for each outcome assessed.	36
**DISCUSSION**			
Discussion	23a	Provide a general interpretation of the results in the context of other evidence.	10-13
	23b	Discuss any limitations of the evidence included in the review.	13
	23c	Discuss any limitations of the review processes used.	13
	23d	Discuss implications of the results for practice, policy, and future research.	10-14
**OTHER INFORMATION**			
Registration and protocol	24a	Provide registration information for the review, including register name and registration number, or state that the review was not registered.	-
	24b	Indicate where the review protocol can be accessed, or state that a protocol was not prepared.	-
	24c	Describe and explain any amendments to information provided at registration or in the protocol.	-
Support	25	Describe sources of financial or non-financial support for the review, and the role of the funders or sponsors in the review.	14-15
Competing interests	26	Declare any competing interests of review authors.	15
Availability of data, code, and other materials	27	Report which of the following are publicly available and where they can be found: template data collection forms; data extracted from included studies; data used for all analyses; analytic code; any other materials used in the	-

Section and Topic	Item #	Checklist item	Location where item is reported
		review.	

Sourced from: Page MJ, McKenzie JE, Bossuyt PM, Boutron I, Hoffmann TC, Mulrow CD, et al. The PRISMA 2020 statement: an updated guideline for reporting systematic reviews. BMJ 2021;372:n71. doi: 10.1136/bmj.n71

2.2. Supplemental Table S2. Meta-analysis of Observational Studies in Epidemiology (MOOSE) checklist.

Item Number	Recommendation	Reported on Page Number
Reporting of background should include		
1	Problem definition	3
2	Hypothesis statement	-
3	Description of study outcome(s)	3
4	Type of exposure or intervention used	3
5	Type of study designs used	-
6	Study population	3
Reporting of search strategy should include		
7	Qualifications of searchers (e.g., librarians and investigators)	-
8	Search strategy, including time period included in the synthesis and key words	4
9	Effort to include all available studies, including contact with authors	-
10	Databases and registries searched	4
11	Search software used, name and version, including special features used (e.g., explosion)	Supplemental Information
12	Use of hand searching (e.g., reference lists of obtained articles)	4
13	List of citations located and those excluded, including justification	-
14	Method of addressing articles published in languages other than English	4
15	Method of handling abstracts and unpublished studies	4
16	Description of any contact with authors	-
Reporting of methods should include		
17	Description of relevance or appropriateness of studies assembled for assessing the hypothesis to be tested	4

18	Rationale for the selection and coding of data (e.g., sound clinical principles or convenience)	5
19	Documentation of how data were classified and coded (e.g., multiple raters, blinding and interrater reliability)	-
20	Assessment of confounding (e.g., comparability of cases and controls in studies where appropriate)	-
21	Assessment of study quality, including blinding of quality assessors, stratification, or regression on possible predictors of study results	Supplemental Information
22	Assessment of heterogeneity	6
23	Description of statistical methods (e.g., complete description of fixed or random effects models, justification of whether the chosen models account for predictors of study results, dose-response models, or cumulative meta-analysis) in sufficient detail to be replicated	5-6
24	Provision of appropriate tables and graphics	26-42
Reporting of results should include		
25	Graphic summarizing individual study estimates and overall estimates.	39-42
26	Table giving descriptive information for each study included	28-30
Reporting of discussion should include		
29	Results of sensitivity testing (e.g., subgroup analysis) Quantitative assessment of bias (e.g., publication bias) Indication of statistical uncertainty of findings	Supplemental Information Information -
30	Justification for exclusion (e.g., exclusion of non-English language citations)	38
31	Assessment of quality of included studies	Supplemental Information
Reporting of conclusions should include		
32	Consideration of alternative explanations for observed results	10-13
33	Generalization of the conclusions (i.e., appropriate for the data presented and within the domain of the literature review)	10-13
34	Guidelines for future research	10-14
35	Disclosure of funding source	14-15

Sourced from: Stroup DF, Berlin JA, Morton SC, et al, for the Meta-analysis Of Observational Studies in Epidemiology (MOOSE) Group. Meta-analysis of Observational Studies in Epidemiology. A Proposal for Reporting. *JAMA*. 2000;283(15):2008-2012. doi: 10.1001/jama.283.15.2008.

2.3. Supplemental Table S3. Methodological quality assessment of included studies using the modified Jadad scale and assessment of funding bias.

Study ID	Criteria 1[a]	Criteria 2[b]	Criteria 3[c]	Criteria 4[d]	Criteria 5[e]	Criteria 6[f]	Criteria 7[g]	Criteria 8[h]	Total MJA Score[i]	Funding Bias[j]
1	0	0	0	0	0	1	1	1	**3**	**1**
2	0	0	0	0	0	1	1	1	**3**	**1**
3	0	0	0	0	0	1	1	1	**3**	**0**
4	0	0	0	0	0	1	1	1	**3**	**1**
5	0	0	0	0	0	1	1	1	**3**	**0**
6	0	0	0	0	0	1	1	1	**3**	**0**
7	1	1	1	0.5	1	1	1	1	**7.5**	**1**
8	1	1	1	0.5	1	1	1	1	**7.5**	**1**
9	0	0	0	0	0	1	1	1	**3**	**0**
10	0	0	1	0.5	0	1	1	1	**4.5**	**0**
11	0	0	0	0	1	1	0	1	**3**	**0**
12	0	0	0	0	0	1	1	1	**3**	**0**
13	0	0	0	0	**1**	1	1	1	**4**	**0**
14	1	1	0.5	1	1	1	1	1	**7.5**	**1**
15	0	0	0	0	0	1	1	1	**3**	**1**
16	0	0	0	0	1	1	1	1	**4**	**0**
17	1	1	1	0.5	1	1	1	1	**7.5**	**1**
18	0	0	1	1	0	1	1	1	**5**	**1**

19	0	0	1	0	0	1	1	1	**4**	**0**
20	0	0	0	0	0	1	1	1	**3**	**1**
21	0	0	0	0	0	1	1	1	**3**	**0**
22	1	1	0.5	1	1	1	1	1	**7.5**	**1**
23	0	0	1	0.5	1	1	1	1	**5.5**	**0**
24	0	0	0	0	0	1	1	1	**3**	**0**
25	0	0	0	0	0	1	1	1	**3**	**0**
26	0	0	0	0	1	1	1	1	**4**	**0**
27	0	0	0	0	0	1	1	1	**3**	**1**
28	0	0	0	0	0	1	0	1	**2**	**1**
29	0	0	1	1	0	1	0	1	**4**	**0**
30	1	1	0.5	1	1	1	1	1	**7.5**	**1**
31	0	0	0	0	1	1	1	1	**4**	**0**
32	0	0	0	0	1	1	1	1	**4**	**1**
33	0	0	0	0	0	0	1	1	**2**	**0**
34	0	0	0	0	0	0	1	1	**2**	**0**
35	1	1	1	0	1	1	1	1	**7**	**1**
36	0	0	0	0	0	1	1	1	**3**	**0**
37	0	0	1	0.5	0	1	1	1	**4.5**	**0**
38	1	1	1	0.5	1	1	1	1	**7.5**	**0**
39	0	0	0	0	0	1	1	1	**3**	**0**
40	0	0	0	0	0	1	1	1	**3**	**0**

41	0	0	0	0	0	1	1	1	**3**	**0**
42	0	0	0	0	0	1	0	1	**2**	**0**
43	0	0	0	0	1	1	0	1	**3**	**0**
44	1	1	1	0.5	1	1	1	1	**7.5**	**0**
45	0	0	1	0.5	1	1	1	1	**5.5**	**1**
46	0	0	0	0	0	1	1	1	**3**	**1**
47	0	0	0	0	0	1	1	1	**3**	**0**
48	0	0	0	0	1	1	1	1	**4**	**0**
49	1	1	0.5	1	1	1	1	1	**7.5**	**1**

Abbreviations: MJA = Modified Jadad Analysis

Note: For all criteria no = 0, yes = 1.

[a]: Criteria 1: Was the study randomised?

:[b] Criteria 2: Was the method of randomisation appropriate?

[c]: Criteria 3: Was the study described as being blinded?

[d]: Criteria 4: Was the method of blinding appropriate? (Single or partially blinded = 0.5)

[e]: Criteria 5: Was there a description of withdrawals and dropouts?

[f]: Criteria 6: Was there a clear description of the inclusion/exclusion criteria?

[g]: Criteria 7: Was the method used to assess adverse events described?

[h]: Criteria 8: Was the method of statistical analysis described?

[i]: Total score = sum of scores across criteria 1-8

[j]: Funding bias: 0=low potential for bias, 1-2=moderate potential for bias (conflicts of interest and/or study funded by industry), 3=high potential for bias (conflicts of interest and industry funding that had a high likelihood of interfering with the study)

2.4. Supplemental Table S4. Outputs from Egger's test for publication bias.

Outcome	Reperfusion Therapy	Std_Eff	Coefficient [95% CI]	Standard Error	t	*P* > I t I	Test of H_0: no small-study effects
Favourable 90-day functional outcomes	**IVT**	**Slope**	-0.50 [-1.08 to 0.07]	0.24	-2.07	0.077	0.625
		**Bias**	-0.76 [-4.28 to 2.76]	1.49	-0.51	0.625	
	**EVT**	**Slope**	-0.53 [-0.92 to -0.14]	0.17	-3.08	0.013	0.162
		**Bias**	1.57 [-0.76 to 3.90]	1.03	1.52	0.162	
sICH	**IVT**	**Slope**	0.45 [0.10 to 0.81]	0.15	2.93	0.019	0.575
		**Bias**	0.30 [-0.88 to 1.47]	0.51	0.58	0.575	
	**EVT**	**Slope**	0.02 [-0.33 to 0.37]	0.15	0.13	0.902	0.777
		**Bias**	-0.14 [-1.24 to 0.96]	0.49	-0.29	0.777	
90-day mortality	**IVT**	**Slope**	0.50 [-0.42 to 1.42]	0.33	1.52	0.204	0.901
		**Bias**	0.22 [-4.47 to 4.92]	1.69	0.13	0.901	
	**EVT**	**Slope**	0.62 [0.28 to 0.97]	0.15	4.14	0.003	0.067
		**Bias**	-1.57 [-3.28 to 0.14]	0.76	-2.08	0.067	

Abbreviations: IVT = intravenous thrombolysis, EVT = endovascular thrombectomy, sICH = symptomatic intracerebral haemorrhage, Std_Eff = standard effect, CI = confidence interval, H_0 = null hypothesis.

3. List of Supplemental Figures

3.1. Supplemental Figure S1. Funnel plots of meta-analyses on the association between atrial fibrillation and clinical outcomes following reperfusion therapy.

3.2. Supplemental Figure S2. Sensitivity analyses for meta-analyses on the association between atrial fibrillation and clinical outcomes following reperfusion therapy.

3.3. Supplemental Figure S3. Forest plots of the association between atrial fibrillation and outcomes following intravenous thrombolysis, stratified by study type.

3.4. Supplemental Figure S4. Forest plots of the association between atrial fibrillation and outcomes following endovascular thrombectomy, stratified by study type.

3.5. Supplemental Figure S5. Forest plots of the pooled prevalence of atrial fibrillation in acute ischaemic stroke patients treated with reperfusion therapy, stratified by study type.

3.6. Supplemental Figure S6. Graphs of Egger's regression tests for the meta-analyses on the association between atrial fibrillation and clinical outcomes following reperfusion therapy.

3.1. Supplemental Figure S1. Funnel plots of meta-analyses on the association between atrial fibrillation and clinical outcomes following reperfusion therapy.

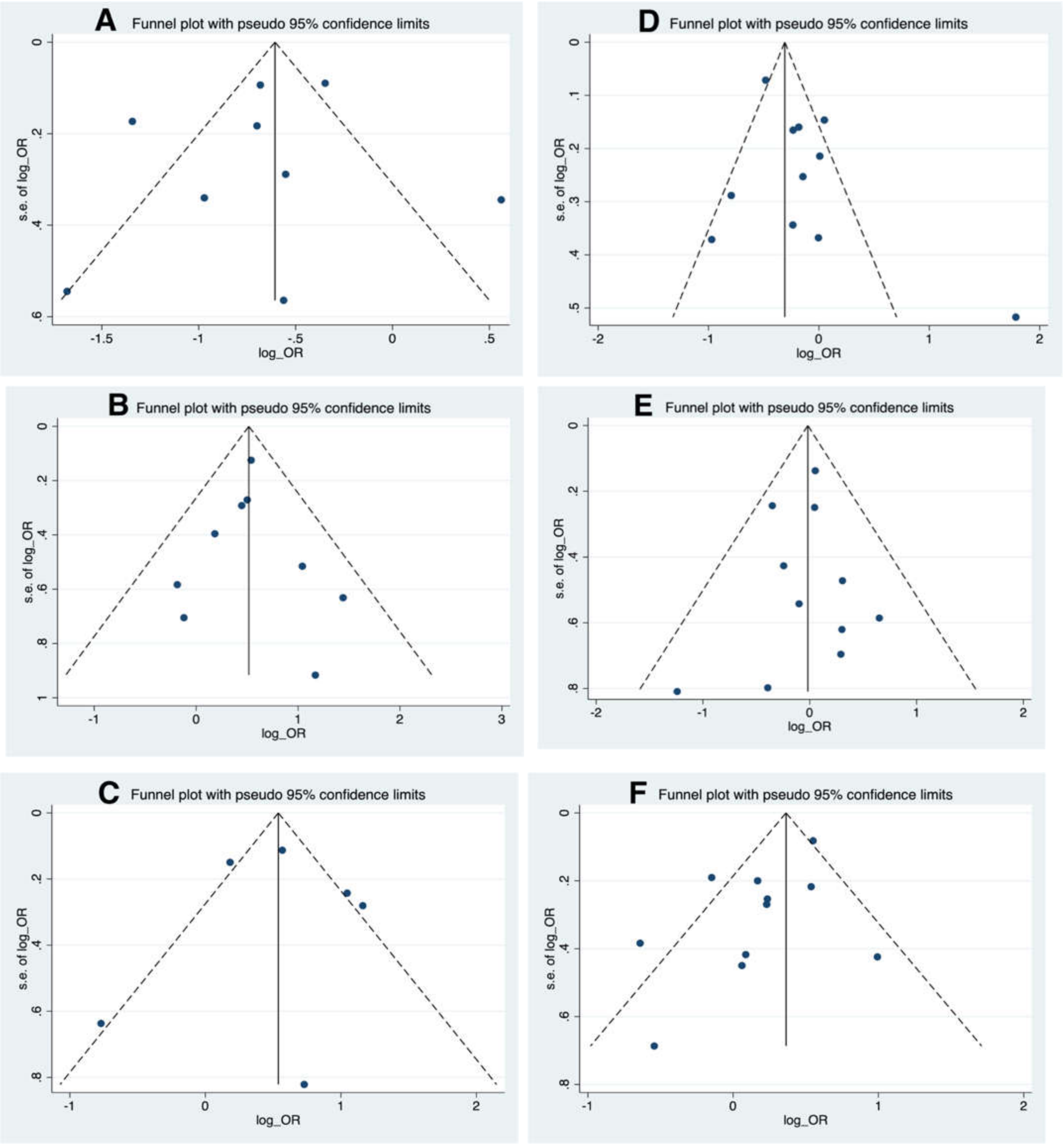

Supplemental Figure S1. Funnel plots of meta-analyses on the association between atrial fibrillation and clinical outcomes following reperfusion therapy.
A: Association between AF and favourable functional 90-day outcomes following IVT. B: Association between AF and sICH following IVT. C: Association between AF and 90-day mortality following IVT. D: Association between AF and favourable functional 90-day outcomes following EVT. E: Association between AF and sICH following EVT. F: Association between AF and 90-day mortality following EVT.
Abbreviations: OR = odds ratio, s.e. = standard error, AF = atrial fibrillation, IVT = intravenous thrombolysis, EVT = endovascular thrombectomy, sICH = symptomatic intracerebral haemorrhage

3.2. **Supplemental Figure S2. Sensitivity analyses for meta-analyses on the association between atrial fibrillation and clinical outcomes following reperfusion**

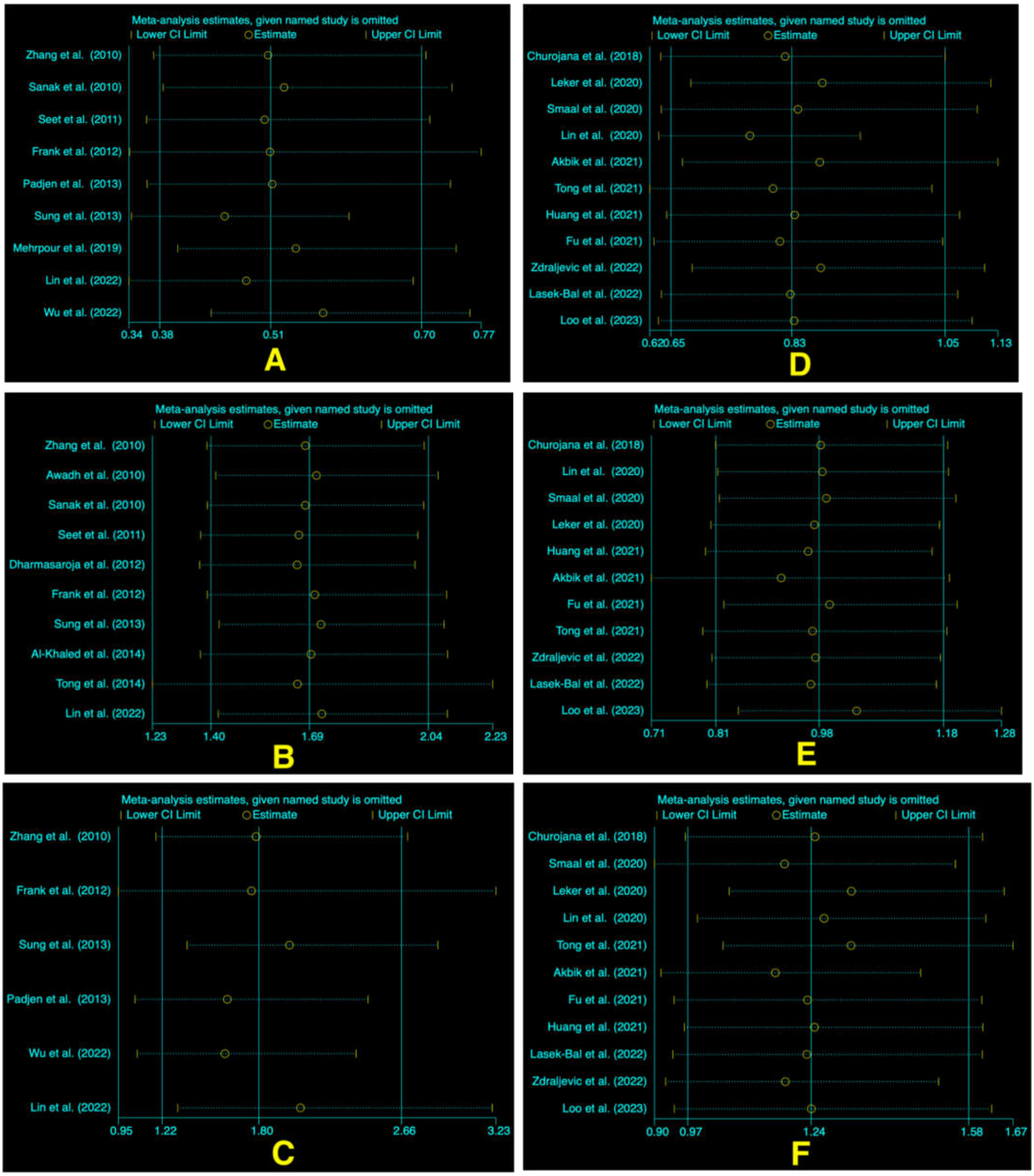

therapy.

Supplemental Figure S2. Sensitivity analyses for meta-analyses on the association between atrial fibrillation and clinical outcomes following reperfusion therapy. A: Association between AF and favourable functional 90-day outcomes following IVT. B: Association between AF and sICH following IVT. C: Association between AF and 90-day mortality following IVT. D: Association between AF and

favourable functional 90-day outcomes following EVT. E: Association between AF and sICH following EVT. F: Association between AF and 90-day mortality following EVT.
Abbreviations: CI = confidence interval

3.3. Supplemental Figure S3. Forest plots of the association between atrial fibrillation and outcomes following intravenous thrombolysis, stratified by study type.

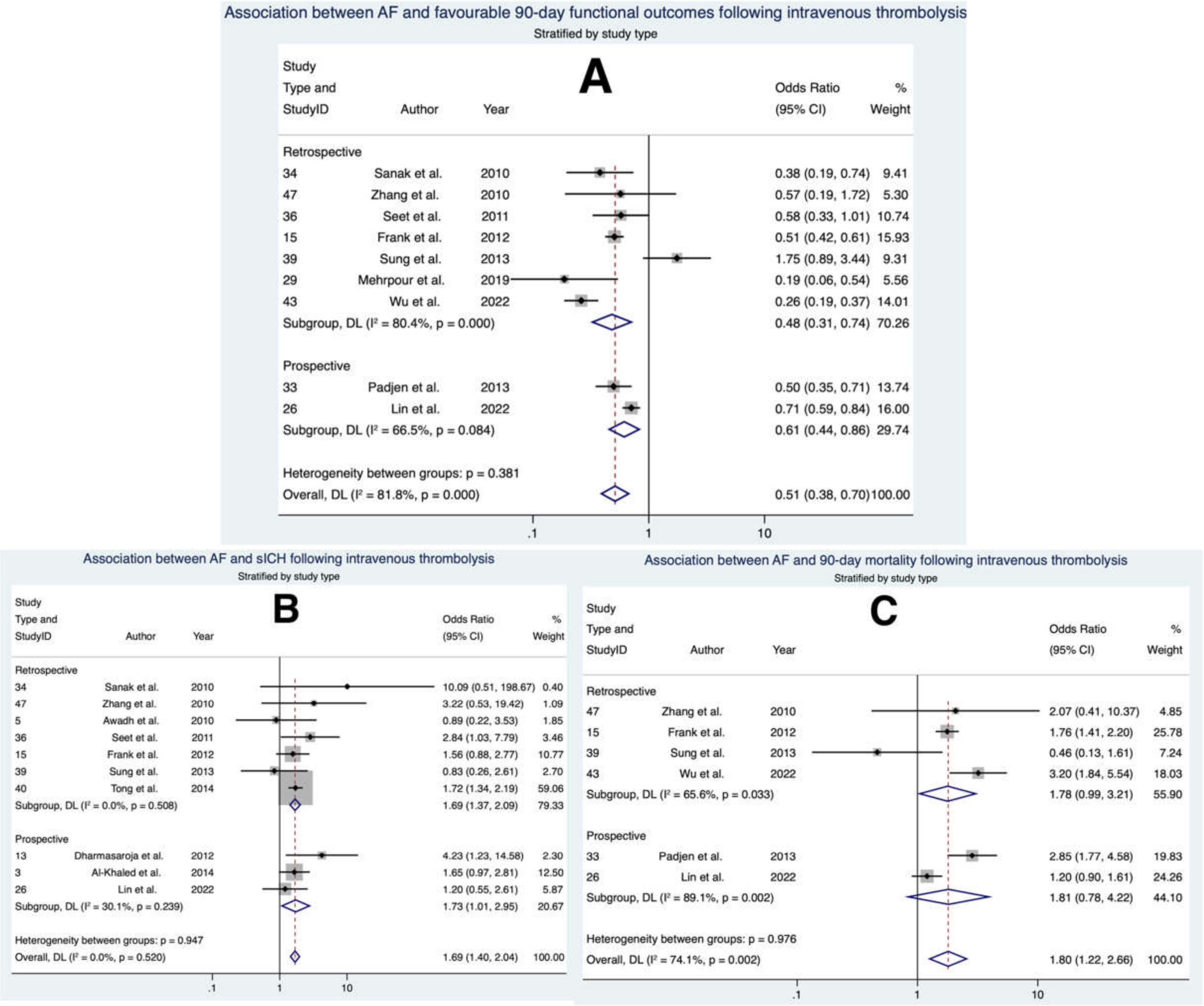

Supplemental Figure S3. Forest plots of the association between atrial fibrillation and outcomes following intravenous thrombolysis, stratified by study type. A: association between atrial fibrillation and favourable 90-day functional outcomes. B: association between atrial fibrillation and symptomatic intracerebral haemorrhage. C: association between atrial fibrillation and 90-day mortality.
Abbreviations: AF = atrial fibrillation, CI = confidence interval, sICH = symptomatic intracerebral haemorrhage, DL = DerSimonian-Laird

3.4. Supplemental Figure S4. Forest plots of the association between atrial fibrillation and outcomes following endovascular thrombectomy, stratified by study type.

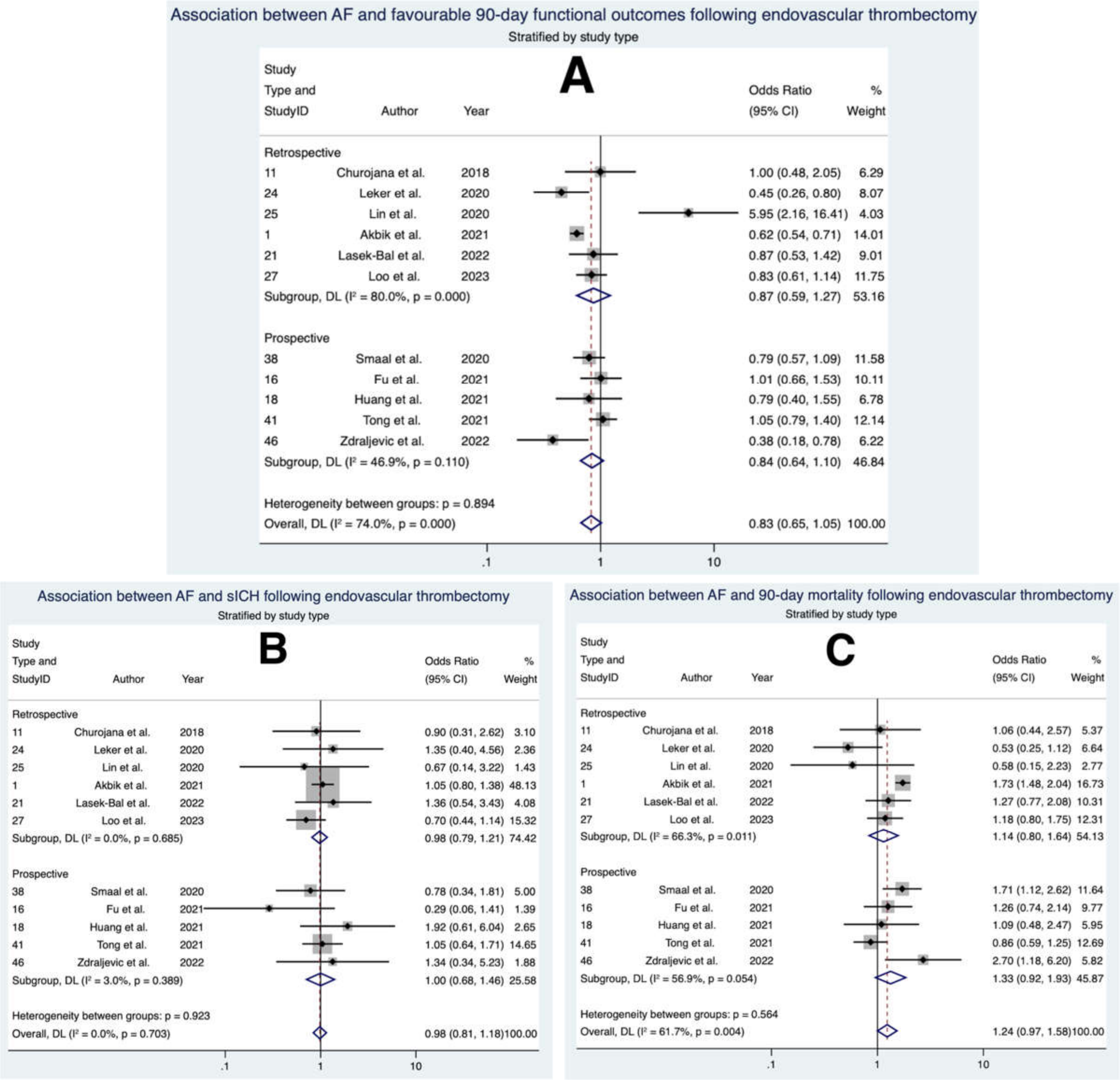

Supplemental Figure S4. Forest plots of the association between atrial fibrillation and outcomes following endovascular thrombectomy, stratified by study type. A: association between atrial fibrillation and favourable 90-day functional outcomes. B: association between atrial fibrillation and symptomatic intracerebral haemorrhage. C: association between atrial fibrillation and 90-day mortality.

Abbreviations: AF = atrial fibrillation, CI = confidence interval, sICH = symptomatic intracerebral haemorrhage, DL = DerSimonian-Laird

3.5. Supplemental Figure S5. Forest plots of the pooled prevalence of atrial fibrillation in acute ischaemic stroke patients treated with reperfusion therapy, stratified by study type.

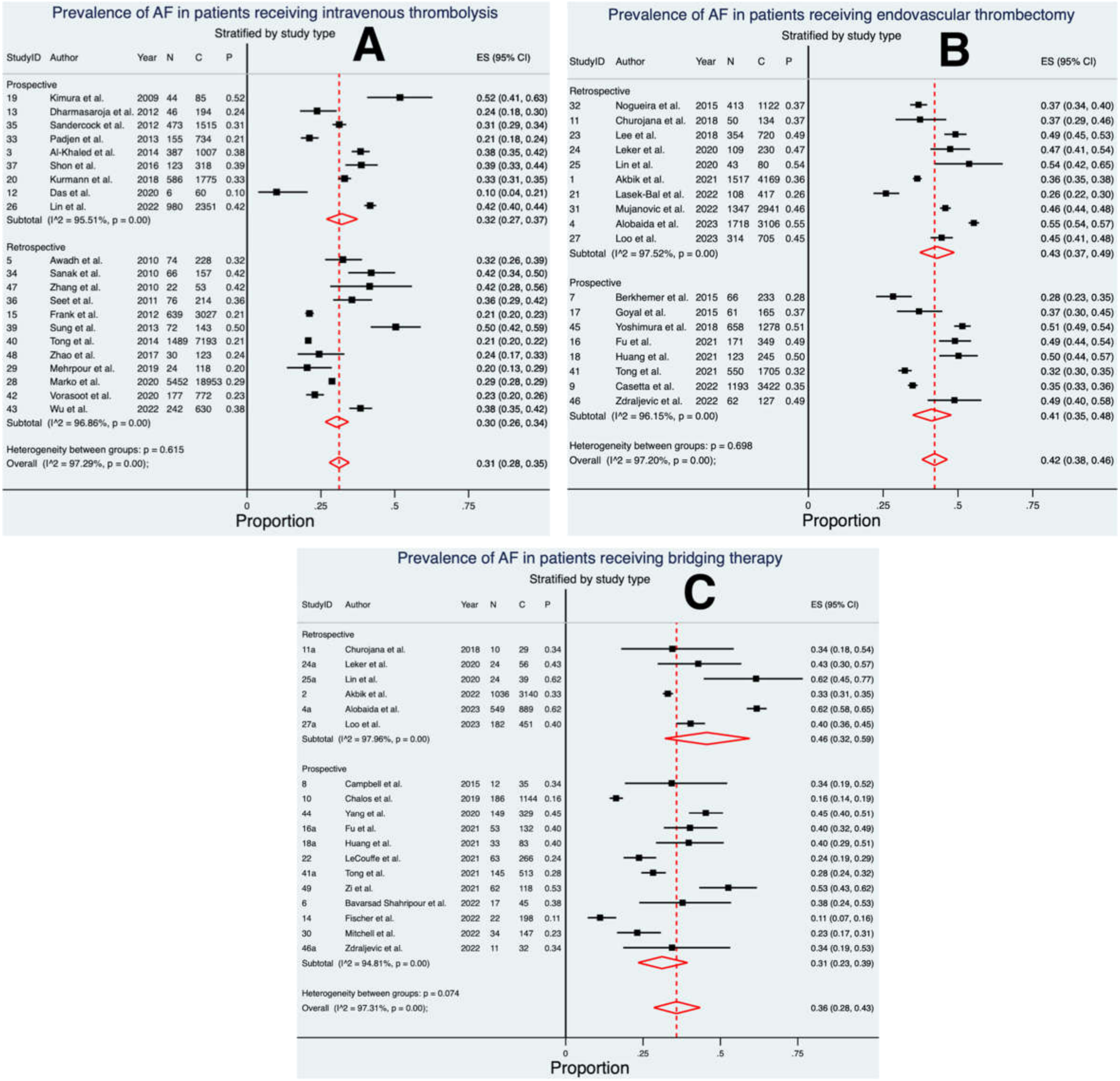

Supplemental Figure S5. **Forest plots of the estimated pooled prevalence of atrial fibrillation in acute ischaemic stroke patients receiving each type of reperfusion therapy, stratified by region. A: intravenous thrombolysis, B: endovascular thrombectomy, C: bridging therapy.**

Abbreviations: AF = atrial fibrillation, ES = effect size, CI = confidence interval, N = number of patients with AF, C = total number of patients, P = prevalence

3.6. Supplemental Figure S6. Graphs of Egger's regression tests for the meta-analyses on the association between atrial fibrillation and clinical outcomes following reperfusion therapy.

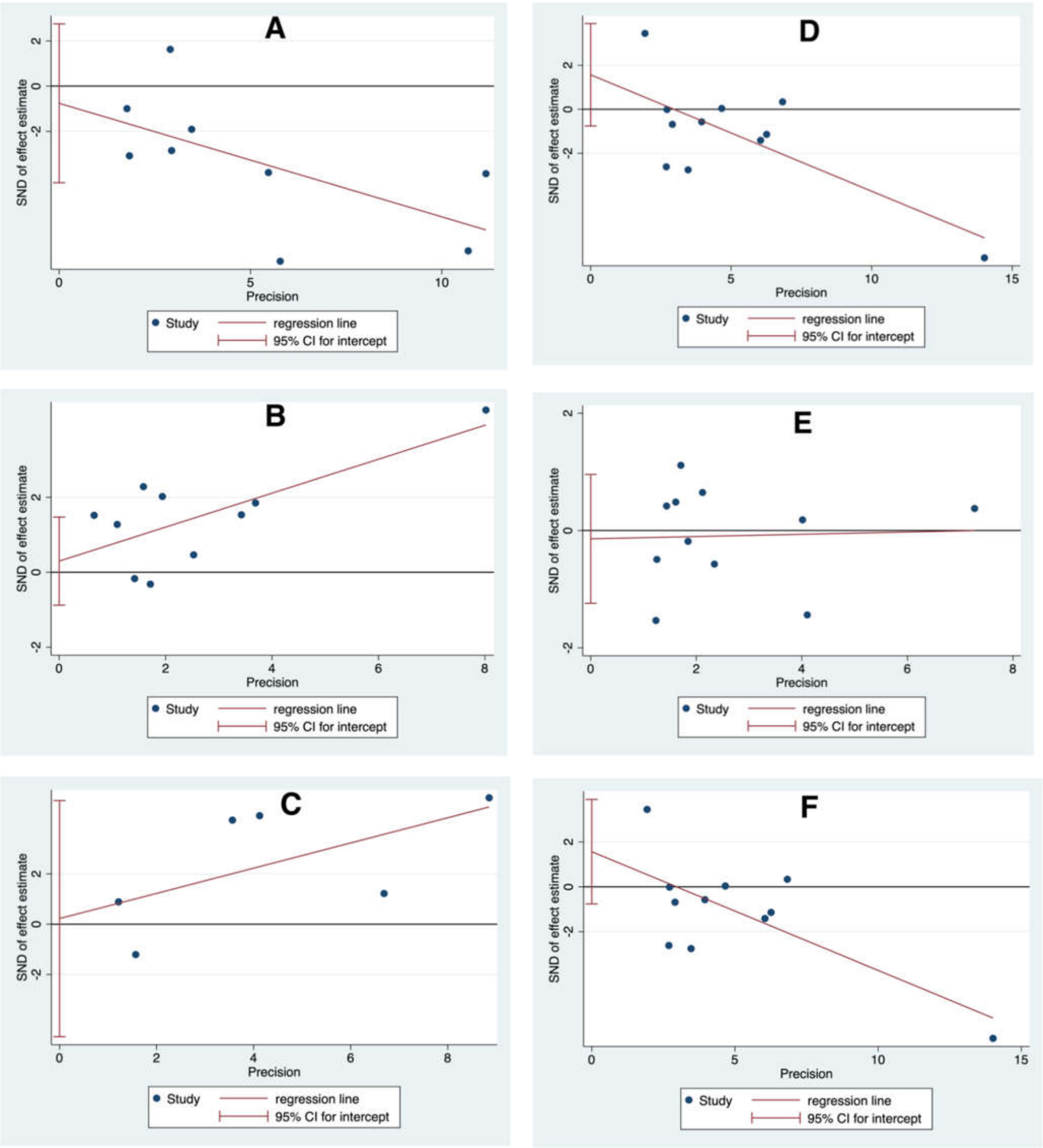

Supplemental Figure S6. Graphs of Egger's regression tests for the meta-analyses on the association between atrial fibrillation and clinical outcomes following reperfusion therapy. A: Association between AF and favourable functional 90-day outcomes following IVT. B: Association between AF and sICH following IVT. C: Association between AF and 90-day mortality following IVT. D: Association between AF and favourable functional 90-day outcomes following EVT. E: Association between AF and sICH following EVT. F: Association between AF and 90-day mortality following EVT.

Abbreviations: CI = confidence interval, SND = standard normal deviate, AF = atrial fibrillation, IVT = intravenous thrombolysis, EVT = endovascular thrombectomy, sICH = symptomatic intracerebral haemorrhage
